# Analytical Strategies for the Detection of Pesticides, Antibiotics, and Heavy Metals in Honey: Current Advances and Implications for Food Safety

**DOI:** 10.3390/foods15162847

**Published:** 2026-08-14

**Authors:** Elena Irina Ursache, Oana Cioanca, Madalina Georgiana Pantazi, Ionut Iulian Lungu, Ioana-Cezara Caba, Ana Flavia Burlec, Andreia Corciova, Monica Hancianu

**Affiliations:** Faculty of Pharmacy, Grigore T. Popa University of Medicine and Pharmacy, 16 University Street, 700115 Iasi, Romania; ursache_elena-irina@d.umfiasi.ro (E.I.U.); madalina-georgiana.pantazi@d.umfiasi.ro (M.G.P.); maria.corciova@umfiasi.ro (A.C.);

**Keywords:** honey contamination, pesticides, heavy metals, veterinary drug residues, emerging contaminants, analytical techniques, LC-MS/MS, biomonitoring, food safety

## Abstract

Honey is a natural food product highly valued for its nutritional and biological properties. Its quality and safety are increasingly affected by environmental contamination and apicultural practices. Among the most relevant contaminants, pesticide residues, veterinary antibiotics, and heavy metals represent major concerns due to their potential impact on human health. This review provides a comprehensive overview of the occurrence, sources, and distribution of these contaminants in honey, with particular emphasis on their relationship with agricultural activities, environmental pollution, and beekeeping treatments. Advanced analytical techniques, including liquid chromatography–tandem mass spectrometry (LC-MS/MS), gas chromatography–mass spectrometry (GC-MS), and inductively coupled plasma–mass spectrometry (ICP-MS), are highlighted for their ability to enable sensitive multi-residue and trace-level detection. The application of chemometric tools for data analysis and sample classification is also addressed, supporting the identification of contamination patterns and origin-related differences. In addition, recent developments in rapid screening methods and environmentally sustainable analytical approaches are discussed. Overall, this review emphasizes the importance of continuous monitoring and the integration of advanced analytical strategies to ensure honey safety, support regulatory compliance, and protect consumers.

## 1. Introduction

Honey is a natural food product recognized for its nutritional value, bioactive composition, and therapeutic properties. Owing to its complex chemical composition and the extensive foraging activity of honeybees, honey also serves as a valuable bioindicator of environmental quality, reflecting contamination originating from agricultural, industrial, and urban ecosystems [1,2,3,4,5,6]. The growing global demand for honey, together with increasing concerns regarding food authenticity and consumer safety, has intensified the need for reliable monitoring of contaminants that may compromise both product quality and public health.

Although honey is generally regarded as a safe and beneficial food, it is susceptible to contamination at multiple stages of production. Environmental pollution, intensive agricultural practices, veterinary treatments applied in apiculture, and inappropriate handling or storage conditions may all contribute to the presence of undesirable chemical residues. Among the contaminants most frequently detected in honey are pesticide residues, veterinary drug residues, heavy metals, and other emerging contaminants, many of which may pose toxicological risks when present above regulatory limits [3,4,5,6].

The unique foraging behavior of *Apis mellifera* enables bees to collect nectar, pollen, water, and plant exudates over large geographical areas, making honey an effective indicator of environmental contamination [7,8]. Pollutants deposited in soil, water, and vegetation may subsequently be transferred into hive products, allowing honey to reflect regional patterns of environmental exposure [9,10]. Consequently, honey has become increasingly important not only as a food commodity but also as a matrix for environmental biomonitoring and food safety assessment [4,11,12,13,14].

Recent advances in analytical chemistry have substantially improved the detection and quantification of contaminants in honey. High-performance chromatographic and spectrometric techniques, including liquid chromatography–tandem mass spectrometry (LC-MS/MS), gas chromatography–mass spectrometry (GC-MS), and inductively coupled plasma–mass spectrometry (ICP-MS), enable highly sensitive multi-residue analysis of diverse contaminant classes. Furthermore, modern sample preparation procedures and chemometric approaches have enhanced analytical performance, facilitating contaminant profiling, geographical discrimination, and authenticity assessment.

This review provides a comprehensive overview of the major chemical contaminants reported in honey, including pesticide residues, veterinary drugs, heavy metals, and emerging contaminants, together with their principal sources, occurrence, toxicological relevance, and regulatory aspects. In addition, recent developments in sample preparation, chromatographic and spectrometric methodologies, chemometric tools, rapid screening strategies, and environmentally sustainable analytical approaches are critically discussed. By integrating current knowledge on contamination pathways with advances in analytical monitoring, this review aims to support future research, strengthen food safety surveillance, and contribute to the production of safer, high-quality honey.

## 2. Literature Search and Bibliometric Overview

A comprehensive literature search was conducted in Scopus to evaluate scientific trends in honey research and contamination. All bibliographic records were retrieved in May 2026, ensuring the inclusion of the most recent indexed publications. Scopus was selected for its extensive multidisciplinary coverage and relevance to bibliometric and scientometric analyses.

**Figure 1:**
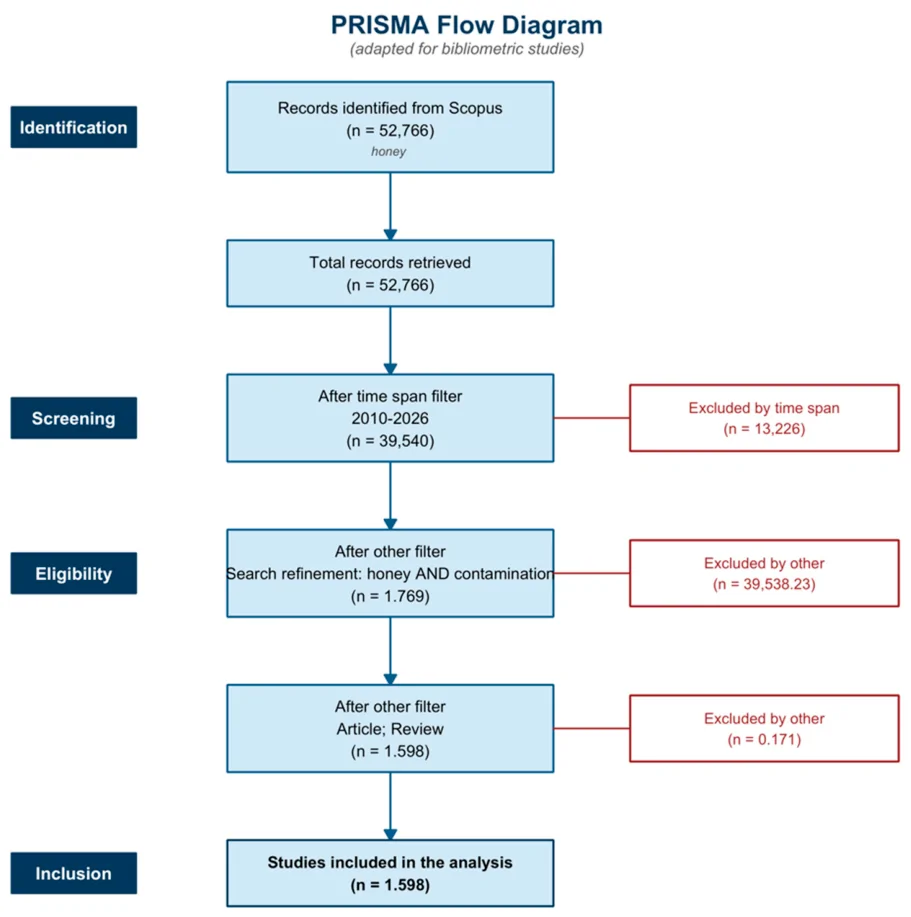
PRISMA flow diagram illustrating the identification, screening, eligibility assessment, and inclusion process used to obtain the final dataset of 1598 publications from the Scopus database. The diagram was generated using the PRISMA module implemented in the Bibliometrix R package (version 5.4.0) and its Biblioshiny web interface based on the filtering criteria applied to the retrieved bibliographic records.

The literature identification, screening, eligibility assessment, and inclusion process used to construct the final bibliometric dataset is summarized in Figure 1.

The initial search was performed using the keyword “honey”. A total of 52,766 documents were identified in the Scopus database. After applying the publication-year filter (2010–2026), 39,540 documents remained, indicating substantial scientific interest in honey-related research in recent years. These publications covered diverse scientific fields, including food science, analytical chemistry, environmental science, agriculture, toxicology, and apicultural research.

To specifically investigate contamination-related studies, a second search strategy was applied using the query “honey AND contamination”. This search retrieved 1769 documents. Additional refinement criteria were subsequently applied to obtain a more relevant and standardized bibliometric dataset. The filtering process included publication years between 2010 and 2026, document types limited to Articles and Reviews, and English-language publications only.

After applying these inclusion criteria, the final bibliometric dataset comprised 1598 scientific publications, which were used in subsequent statistical and bibliometric analyses. The collected bibliographic information included publication year, authors, affiliations, source titles, abstracts, author keywords, DOI information, and citation data. The exported database was analyzed in Microsoft Excel to produce descriptive statistics and graphical visualizations of publication trends. In addition, bibliometric mapping, keyword co-occurrence analysis, and thematic evolution analysis were performed using the Bibliometrix R package and its Biblioshiny web interface to identify major research themes, conceptual structures, scientific trends, and emerging analytical directions in honey contamination research.

While all 1598 publications were included in the bibliometric analysis, only a representative subset was chosen for citation and detailed discussion in the review’s narrative sections. Priority was given to recent studies directly related to the contaminants and analytical methods discussed, as well as to key publications demonstrating methodological advances, analytical applications, and current challenges. This selection aimed to keep the scope focused and manageable, and does not suggest that the remaining publications are of lesser scientific importance. Instead, the cited studies serve as representative examples that best support the specific topics critically examined in this review.

### 2.1. Scopus Database Analysis

**Figure 2:**
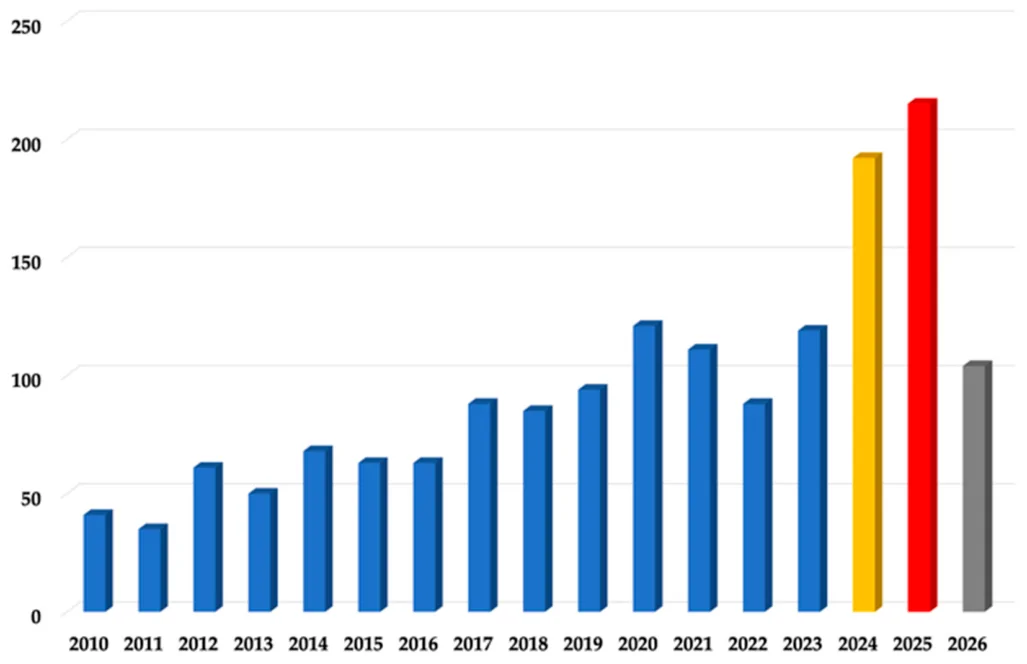
Annual scientific publications on honey contamination indexed in Scopus (2010–2026).

The bibliometric evaluation showed a steady increase in the number of scientific publications on honey contamination during the period under review. A significant growth trend was particularly observed after 2017, with the highest publication outputs recorded in 2024 and 2025. This increase reflects growing global concerns about food safety, environmental contamination, pesticide residues, veterinary drug monitoring, heavy metal accumulation, and the development of advanced analytical methodologies for detecting contaminants in honey and other bee products. Figure 2 illustrates the annual number of scientific publications related to honey contamination indexed in the Scopus database between 2010 and 2026.

### 2.2. Distribution of Publications by Subject Area

The bibliometric analysis showed that research on honey contamination is highly multidisciplinary, spanning multiple scientific fields, including food safety, environmental monitoring, analytical chemistry, toxicology, and biological sciences. The highest number of publications was recorded in Agricultural and Biological Sciences (779 documents), underscoring the strong connection between research on honey contamination and topics such as apicultural production, farming practices, environmental exposure, and bee health.

Chemistry was the second most relevant subject area, with 616 publications, reflecting the extensive use of advanced analytical techniques for contaminant detection and quantification. Environmental Science also contributed substantially (430 publications), demonstrating growing scientific interest in environmental pollutants, pesticide residues, heavy metals, and ecological contamination pathways affecting honey and bee products.

Key research areas also included Biochemistry, Genetics, and Molecular Biology (261 publications), Medicine (239 publications), and Pharmacology, Toxicology, and Pharmaceutics (232 publications). These fields highlight an increasing focus on toxicological testing, biomonitoring, food safety, and the exploration of human health risks linked to contaminated honey.

Although smaller in scale, notable contributions have emerged in the fields of Immunology and Microbiology, Engineering, Chemical Engineering, and Veterinary Sciences. This underscores the expanding scope of interdisciplinary approaches encompassing biosensors, nanotechnology, microbiological safety assessment, veterinary drug surveillance, and sophisticated analytical platforms for contaminant detection.

**Figure 3:**
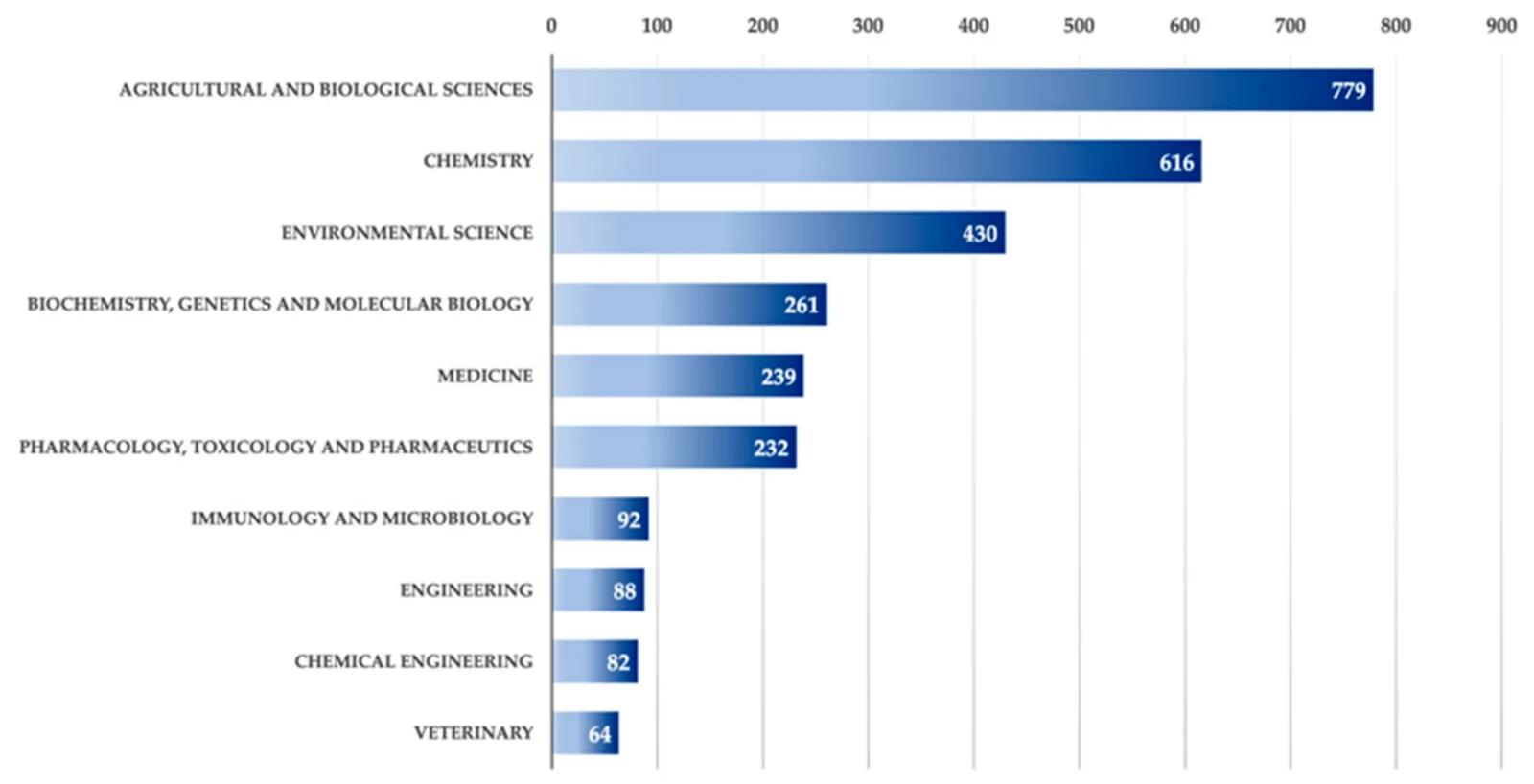
Subject area distribution of scientific publications related to honey contamination indexed in the Scopus database (2010–2026).

Overall, the publication distribution by subject area shows that honey contamination research is a multifaceted, multidisciplinary field involving agricultural sciences, analytical chemistry, environmental monitoring, toxicology, and biomedical research. Figure 3 displays the distribution of scientific publications across the key Scopus subject areas.

### 2.3. Bibliometric Mapping Using Bibliometrix/Biblioshiny

Bibliometric and science mapping analyses were conducted using the R package bibliometrix and its web-based interface Biblioshiny. The analysis was performed in the R statistical environment (version 4.6) using bibliographic data exported from the Scopus database in BibTeX format.

The final dataset comprised 1598 scientific publications related to honey contamination, environmental pollutants, pesticides, heavy metals, antibiotics, microplastics, and bee health. Bibliometric analyses were used to identify the main research trends, conceptual relationships, and thematic evolution within the scientific literature.

To investigate the conceptual structure of the research field, a keyword co-occurrence network was constructed from author keywords. The network was constructed using association normalization and the Louvain clustering algorithm. Isolated nodes were removed to improve interpretability, and the visualization was limited to the 20 most relevant keywords.

**Figure 4:**
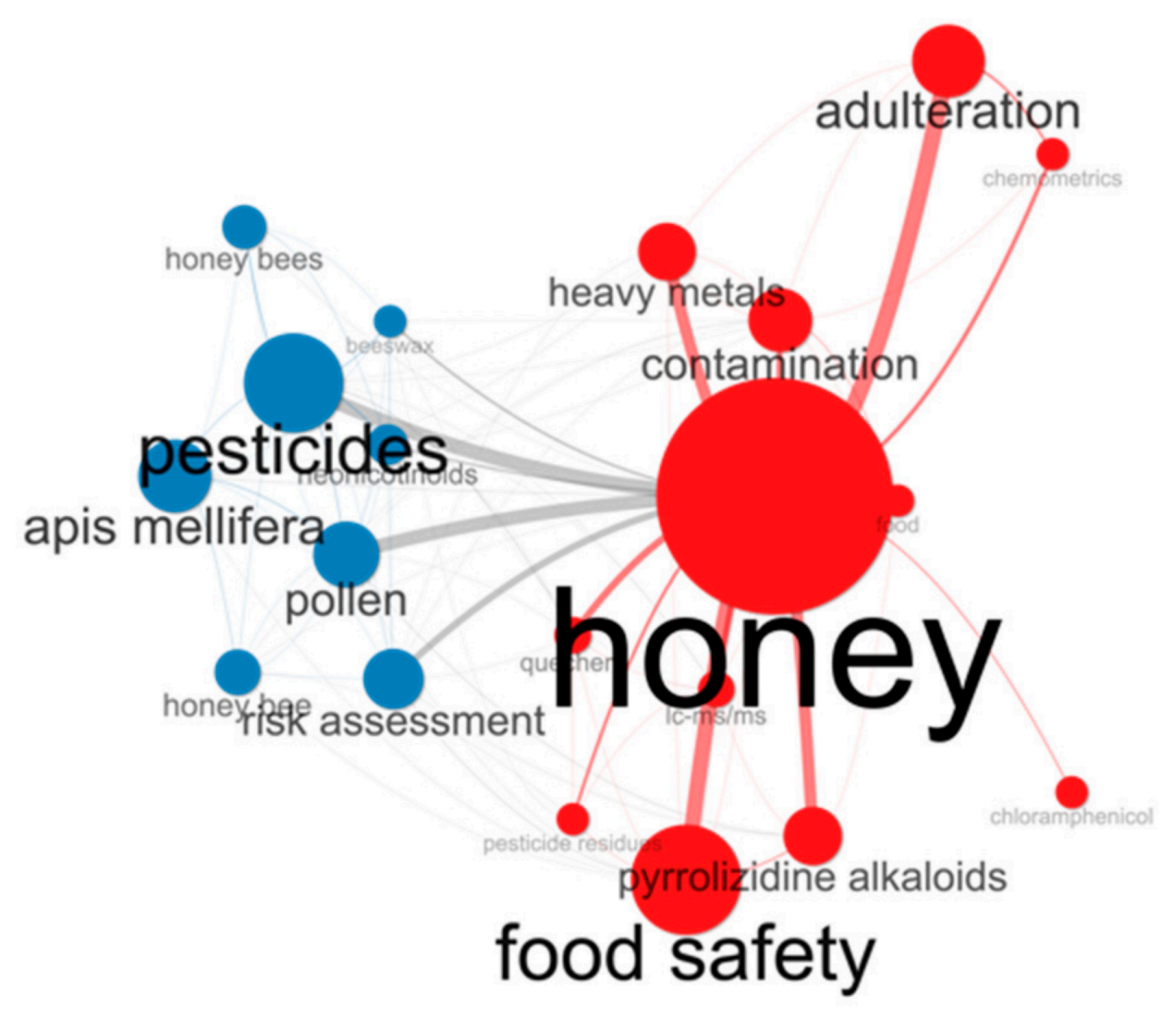
Conceptual structure of the scientific literature on honey contamination and environmental pollutants based on keyword co-occurrence analysis using Bibliometrix/Biblioshiny.

As shown in Figure 4, the co-occurrence network revealed three major thematic clusters. The first cluster (red) was centered on honey contamination, food safety, adulteration, heavy metals, pyrrolizidine alkaloids, pesticide residues, and analytical approaches such as LC–MS/MS and chemometrics. This cluster reflects the strong emphasis on contaminant detection, honey authenticity, food quality, and analytical monitoring.

The second cluster (blue) was primarily associated with honey bees, pesticides, neonicotinoids, pollen, residues, *Apis mellifera*, and risk assessment. The close connections among these keywords indicate the growing scientific interest in pesticide exposure and its ecological and toxicological effects on pollinator health.

The third cluster (green) focused on environmental contamination issues, including microplastics, pollution, contamination, heavy metals, and bee products. This cluster highlights the increasing use of honey and bee-derived products as indicators of environmental quality and pollution monitoring.

Furthermore, thematic evolution analysis was conducted using the Bibliometrix/Biblioshiny framework to investigate the temporal progression of major research themes in the scientific literature. The analysis was based on author keywords and included 250 terms, with a minimum cluster frequency of 5 per thousand documents. Thematic links were filtered using a minimum weight index of 0.2, while the α parameter, which balances occurrence and centrality, was set to 0.5. The Louvain clustering algorithm was applied with a label size of 0.3 and 30 labels displayed per cluster. Temporal evolution was divided into four periods using three cutting points (2010, 2016, and 2022), resulting in the time slices 2010–2010, 2011–2016, 2017–2022, and 2023–2026.

**Figure 5:**
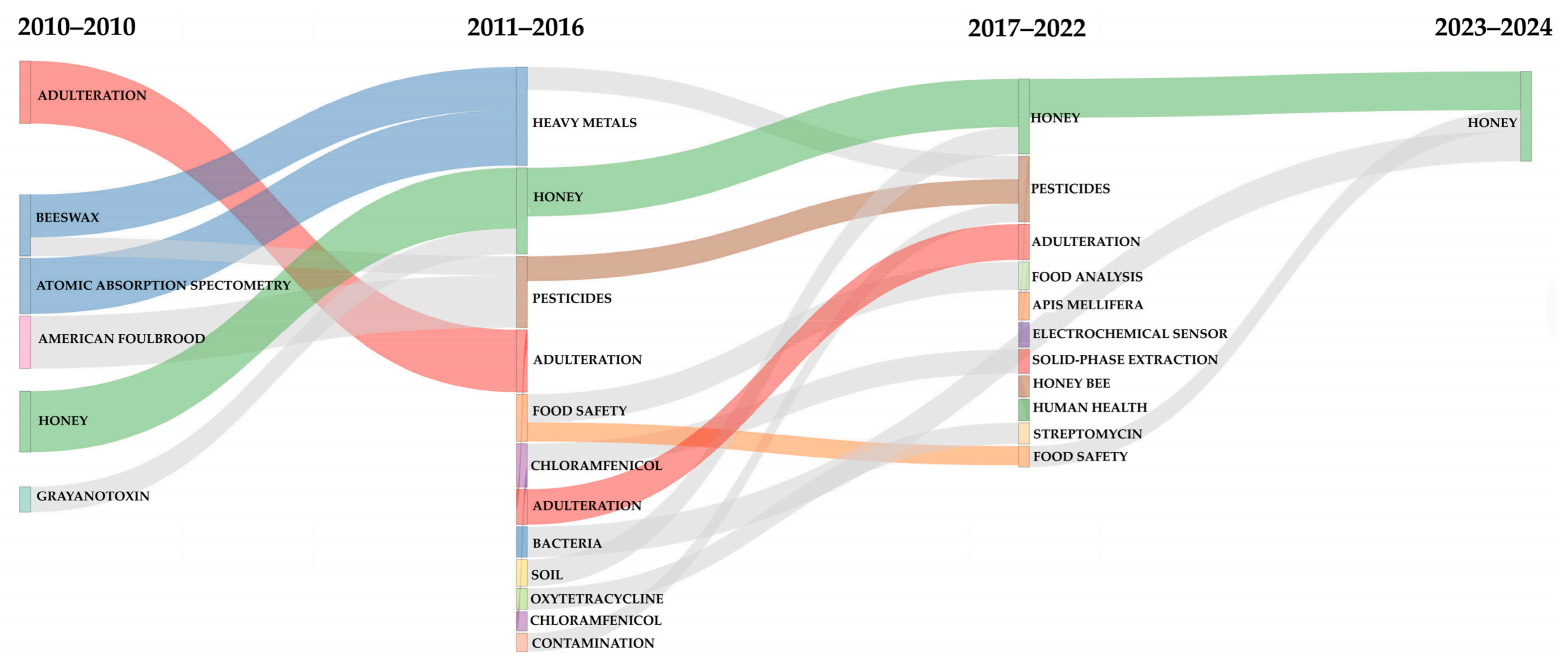
Thematic evolution of research topics related to honey contamination and environmental pollutants generated using the Bibliometrix/Biblioshiny framework.

As illustrated in Figure 5, the initial research predominantly focused on honey authenticity, adulteration, beeswax, and analytical methodologies. Over subsequent periods, the research emphasis gradually shifted towards contamination, pesticides, food safety, and environmental monitoring. Recently, emerging themes such as pesticide exposure, honeybee health, electrochemical sensing technologies, and environmental contamination have gained prominence. The persistent occurrence of the keyword “honey” across all time periods underscores its pivotal role in connecting various interdisciplinary research domains.

**Figure 6:**
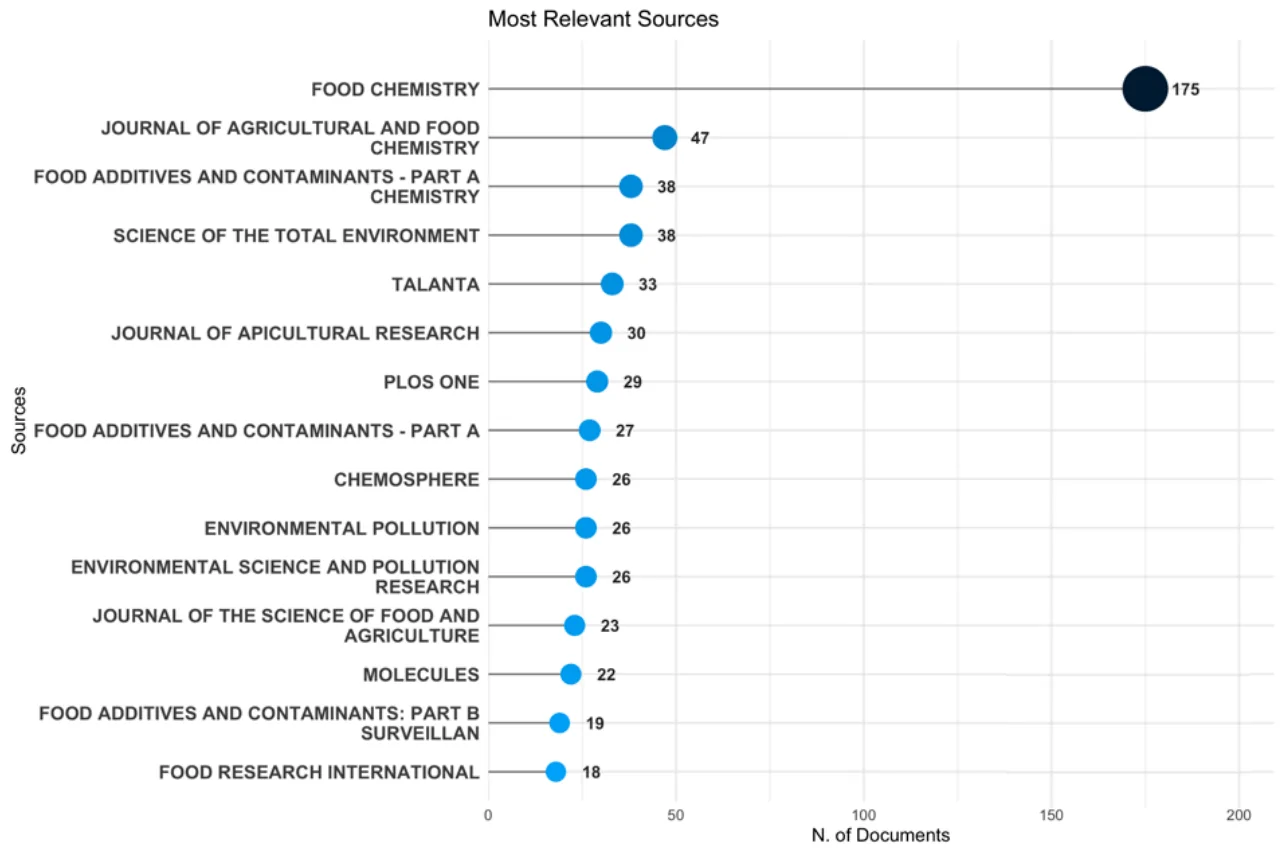
Most relevant scientific journals publishing research related to honey contamination and environmental pollutants based on Bibliometrix/Biblioshiny source analysis.

Additionally, the key scientific sources contributing to honey contamination research were identified using the Bibliometrix/Biblioshiny framework. As presented in Figure 6, Food Chemistry was the leading publication source, with 175 documents, substantially exceeding all other journals in the dataset. Other highly productive journals included Journal of Agricultural and Food Chemistry, Food Additives and Contaminants, Science of the Total Environment, Talanta, and Journal of Apicultural Research. The strong representation of journals related to food chemistry, environmental sciences, analytical chemistry, and toxicology highlights the interdisciplinary nature of honey contamination research. In particular, the prominence of analytical and environmental journals indicates the increasing scientific focus on contaminant detection, food safety assessment, biomonitoring, and environmental pollution associated with honey and bee-related products.

**Figure 7:**
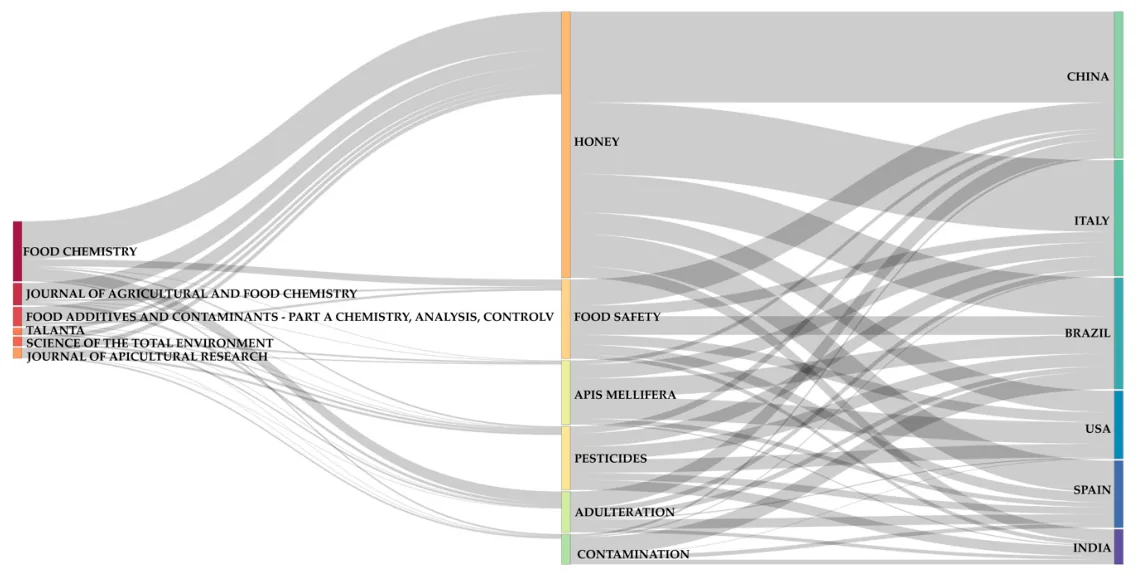
Three-fields plot showing the relationships among the most relevant scientific sources (**left field**), author keywords (**middle field**), and contributing countries (**right field**) in the scientific literature on honey contamination and environmental pollutants, generated using Bibliometrix/Biblioshiny.

As shown in Figure 7, the three-fields plot highlights the relationships among the most influential scientific journals, the most frequently used author keywords, and the leading contributing countries in honey contamination research. The keyword “honey” emerges as the dominant thematic hub, linking major publication sources such as Food Chemistry, Journal of Agricultural and Food Chemistry, Food Additives and Contaminants—Part A, Talanta, Science of the Total Environment, and Journal of Apicultural Research with the most productive countries, including China, Italy, Brazil, the United States, Spain, and India. Additional prominent themes, namely food safety, *Apis mellifera*, pesticides, adulteration, and contamination, reflect the multidisciplinary nature of research in this field, encompassing food quality and authenticity assessment, environmental contamination, analytical chemistry, and bee health. Overall, the visualization demonstrates the strong international research interest in honey contamination and related environmental pollutants, while emphasizing the central role of honey as the primary research focus connecting journals, topics, and countries.

**Figure 8:**
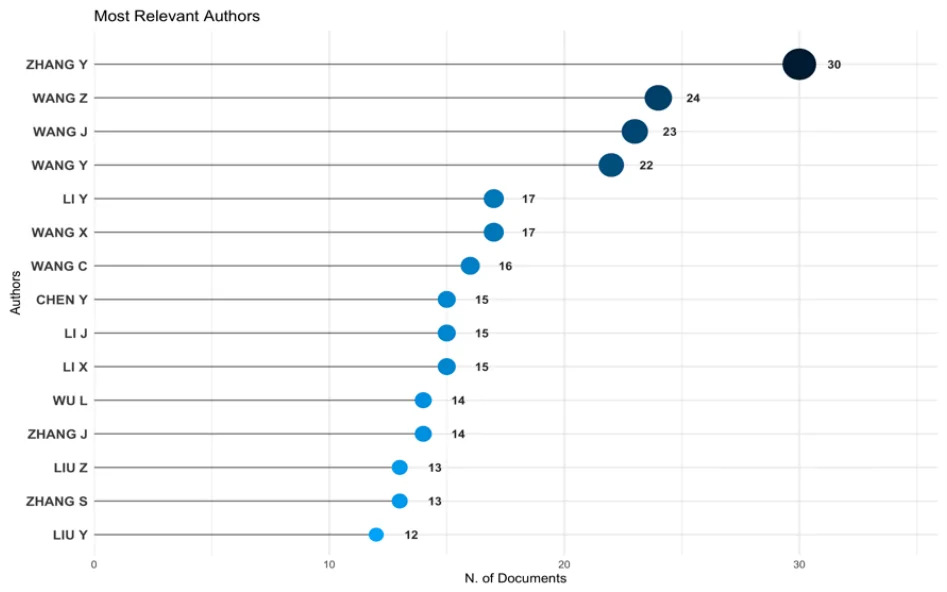
Most productive scientific authors contributing to research on honey contamination and environmental pollutants based on Bibliometrix/Biblioshiny analysis.

The most productive scientific authors involved in honey contamination research were identified using the Bibliometrix/Biblioshiny framework. As shown in Figure 8, the top authors primarily focused on themes including honey contamination, pesticide residues, food safety, environmental pollutants, analytical methods, and bee health. Notably, Zhang Y, Wang Z, Wang J, and Wang Y stood out with the highest publication productivity, indicating ongoing research in contaminant detection, environmental monitoring, and honey quality. The predominance of authors from Asian institutions, especially Chinese researchers, suggests growing global research efforts and expertise in honey safety and contamination monitoring.

Overall, the bibliometric analysis revealed a continuous expansion and increasing diversification of scientific research on honey contamination. The integration of publication trends, conceptual mapping, thematic evolution, scientific sources, author productivity, and interdisciplinary keyword relationships demonstrated that research on honey contamination has evolved into a highly multidisciplinary field that integrates food safety, analytical chemistry, toxicology, environmental monitoring, and pollinator health. Furthermore, the persistence of central themes related to contamination, pesticide residues, environmental pollutants, and honeybee health highlights the growing scientific interest in understanding contaminant pathways, improving analytical detection strategies, and ensuring honey quality and consumer safety.

## 3. Sources and Types of Contaminants in Honey

Honey contamination is a complex issue resulting from both natural processes and anthropogenic activities throughout the production chain. Environmental pollution, intensive agricultural practices, veterinary treatments in apiculture, and improper hive management or post-harvest handling can all contribute to chemical contaminants in honey [15,16,17]. Because honeybees forage over large geographic areas and continuously interact with environmental resources, honey reflects contaminant exposure from surrounding ecosystems and serves as an important matrix for food safety assessment and environmental biomonitoring [18,19,20,21].

The occurrence of contaminants in honey originates from both natural and human activities, either directly through beekeeping practices or indirectly via environmental exposure to contaminated floral and water sources used by honeybees [11,22,23]. Pollutants from industry, agriculture, traffic, pesticides, atmospheric fallout, and waste disposal can accumulate in bee products, affecting honey’s composition, quality, and safety [10]. Moreover, contamination can result from poor hive management, incorrect post-harvest handling, inadequate storage, or economic adulteration, all of which threaten product authenticity and consumer confidence [1].

**Figure 9:**
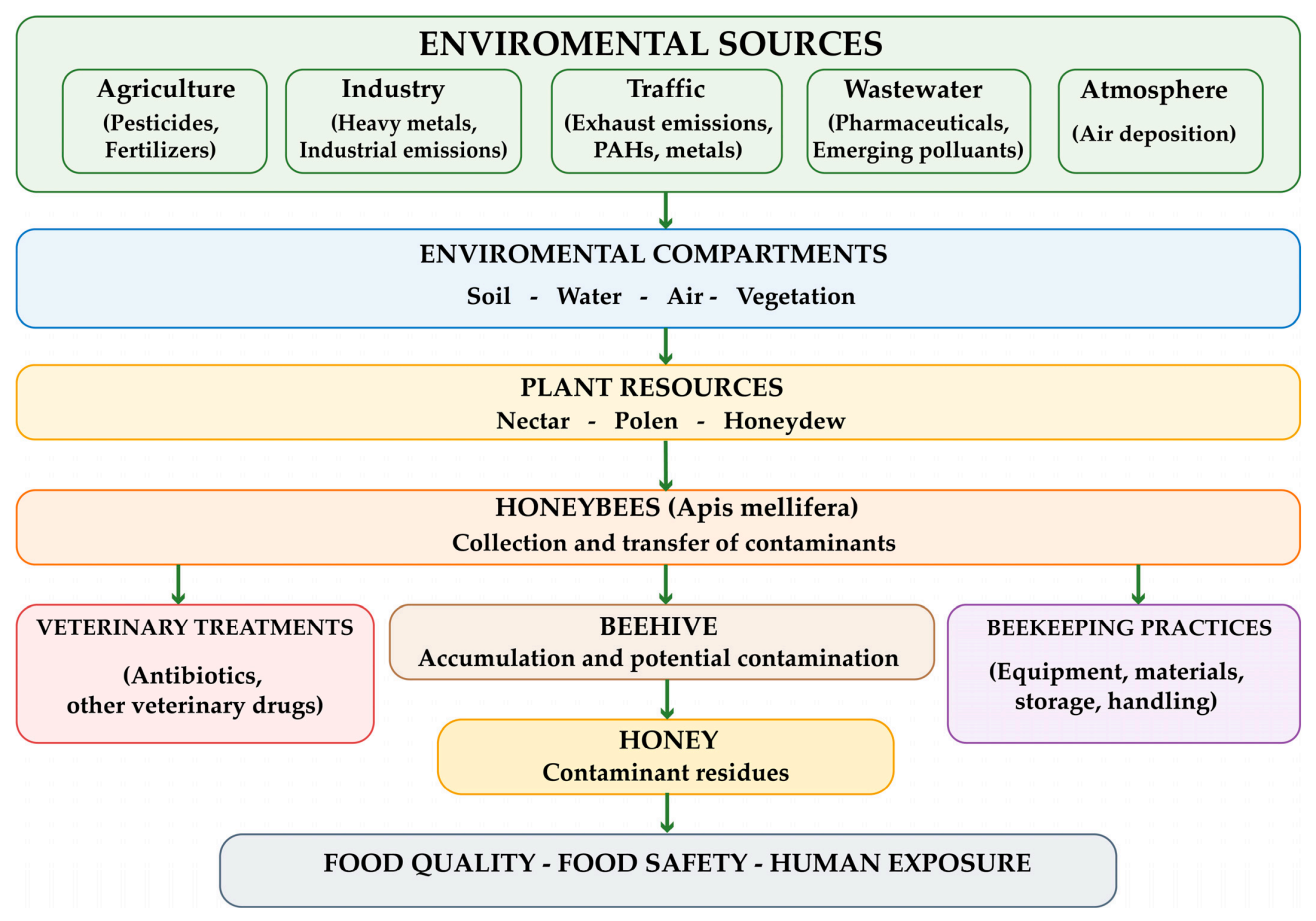
Major sources and pathways of contaminant transfer into honey.

Due to honey’s nutritional, therapeutic, and economic value, honey safety has become increasingly important to scientists and regulators worldwide. Contaminants in honey can negatively impact product quality, authenticity, pollinator health, ecosystems, food safety, and human health [1,12]. Additionally, as the international honey trade grows, strict monitoring and standardized quality assurance are vital to meet food safety standards and residue limits set by various authorities [12]. Understanding contamination sources, how they occur, and their toxicological effects is crucial for ensuring honey safety, environmental monitoring, and public health protection. The main sources of contaminants and their pathways of transfer into honey are summarized in Figure 9.

### 3.1. Pesticides

Pesticide residues are among the most extensively studied contaminants in honey due to the intensive and widespread use of pesticides in modern agriculture. These compounds are used to control pests, boost agricultural yields, and safeguard crops. However, only a small amount reaches the intended biological targets, while a large portion disperses into nearby ecosystems, impacting non-target organisms such as pollinators [24].

Honeybees are exposed to pesticides mainly during foraging activities through the collection of contaminated nectar, pollen, water, and airborne particles from treated plants and agricultural landscapes. As a result, pesticide residues may be transferred into hive products, including honey, wax, pollen, and propolis [24,25]. Numerous studies conducted across different geographical regions have reported pesticide residues in honey samples, confirming the widespread occurrence of contamination even in products originating from apparently uncontaminated environments [14,16,17,21].

In recent years, concerns about honey quality and honeybee health have increased, primarily due to environmental stressors such as habitat destruction, declining bee colonies, nutritional deficiencies, pests, diseases, and contamination from agricultural practices [25]. Among these, the extensive use of pesticides on crops and pollination sources is a significant factor contributing to honey contamination. This emphasizes the necessity for continuous surveillance and rigorous analytical procedures to ensure honey safety and to protect the health of both bees and humans [25,26].

Several pesticide classes have been identified in honey, including organophosphates, organochlorines, carbamates, pyrethroids, and neonicotinoids [27]. Among these, neonicotinoids have attracted particular scientific attention due to their systemic properties, persistence, and neurotoxic effects on pollinators. These compounds act on nicotinic acetylcholine receptors, affecting bee orientation, memory, feeding behavior, immune response, and survival, even at low concentrations [26]. In addition, persistent organic pollutants (POPs), including polychlorinated biphenyls (PCBs) and polybrominated diphenyl ethers (PBDEs), have also been reported in honey, reflecting long-term environmental contamination and atmospheric transport mechanisms [26].

The occurrence and concentration of pesticide residues in honey are influenced by several factors, including agricultural intensity, crop type, application frequency, environmental persistence, climatic conditions, and seasonal agricultural practices [24,28]. Variability in pesticide contamination has also been associated with differences in regional pesticide use patterns, environmental transport processes, and the persistence of residues in soil, water, and vegetation, which may subsequently influence bee exposure during nectar and pollen collection [28]. Although pesticide concentrations are frequently reported at trace levels ranging from µg/kg to low mg/kg and generally remain below established maximum residue limits (MRLs), elevated concentrations have occasionally been reported, particularly in highly intensive agricultural areas [14,21,26].

Beyond food contamination concerns, pesticide residues in honey have important toxicological implications. Long-term exposure to pesticides has been associated with endocrine disruption, neurotoxicity, oxidative stress, reproductive disorders, carcinogenicity, and immunological alterations [2,26]. Consequently, continuous analytical monitoring of pesticide residues remains essential for ensuring honey safety, environmental sustainability, and consumer protection.

While significant strides have been made in detecting pesticide residues in honey, some challenges persist. Advanced chromatographic techniques such as LC-MS/MS and GC-MS have greatly enhanced the ability to detect multiple pesticide classes at trace levels simultaneously. However, the ongoing emergence of new active ingredients, the presence of complex multi-residue mixtures, and variations among national monitoring programs still hinder data comparison and risk assessment. Future efforts should aim to standardize analytical methods, broaden multi-residue screening, and better evaluate combined exposure to pesticide mixtures.

### 3.2. Veterinary Antibiotics

Veterinary antibiotics represent another important class of contaminants detected in honey and are mainly associated with apicultural management practices. These compounds are commonly used in beekeeping to prevent and treat bacterial diseases affecting bee colonies, particularly American foulbrood (*Paenibacillus larvae*) and European foulbrood (*Melissococcus plutonius*) [29,30]. Although the use of antibiotics in apiculture is highly regulated or prohibited in several countries, especially within the European Union, residues continue to be reported in honey samples, suggesting inappropriate use, illegal treatments, environmental contamination, or insufficient withdrawal periods [31].

The transfer of antibiotic residues into honey may occur directly through hive treatments or indirectly through contamination of hive materials and environmental sources. Once introduced into beekeeping systems, these substances may persist within hive matrices such as wax, pollen, and propolis and subsequently migrate into honey during production and storage [31]. Furthermore, cross-contamination during honey processing, storage, or transportation may contribute to residue formation and complicate the understanding of contamination pathways [31].

Several antibiotic classes have been reported in honey, including tetracyclines, sulfonamides, macrolides, quinolones, aminoglycosides, amphenicols, nitrofurans, and lincosamides, reflecting both therapeutic applications in apiculture and environmental exposure pathways [32,33,34]. Among these compounds, tetracyclines, macrolides, and sulfonamides are among the most frequently monitored due to their historical use in the prevention and treatment of bacterial diseases affecting honeybee colonies, particularly American and European foulbrood [33,34]. Studies have shown that residue persistence in honey varies considerably with the antibiotic’s physicochemical properties, treatment practices, and hive conditions, with some compounds remaining detectable for extended periods after administration [34].

The occurrence and concentration of antibiotic residues in honey are influenced by multiple factors, including veterinary regulations, beekeeping management, environmental contamination, withdrawal periods, and monitoring strategies [32,33]. Although residue levels are often detected at trace concentrations, the presence of these compounds in honey remains a food safety concern because prolonged exposure may contribute to antimicrobial resistance, allergic reactions, and other adverse public health effects [32]. Furthermore, the lack of harmonized maximum residue limits for several antibiotics in honey complicates residue control and underscores the importance of continuous surveillance and sensitive analytical methods for detecting residues [31,34].

Another important aspect concerns the use of acaricides to control parasitic infestations, particularly those caused by *Varroa destructor*. Although these substances are primarily intended to control mites rather than bacterial infections, certain acaricides may accumulate in hive products and indirectly contribute to honey contamination, thereby increasing the chemical complexity of hive matrices and analytical challenges associated with residue determination [24].

The presence of antibiotic residues in honey remains a food safety concern because inappropriate or uncontrolled use, inadequate withdrawal periods, and environmental exposure may contribute to residue accumulation, thereby increasing the risk of antimicrobial resistance and other adverse public health outcomes [32,34]. Given these concerns, the implementation of strict veterinary control measures, residue monitoring strategies, and good beekeeping practices remains fundamental for minimizing contamination risks and maintaining consumer confidence in honey quality and authenticity.

Recent progress in elemental analysis, especially ICP-MS methods, has greatly enhanced the precision of trace metal detection in honey. Nonetheless, key challenges persist, such as the absence of standardized monitoring approaches, variations in contamination levels across different regions, and limited insight into long-term environmental trends. Future research should combine analytical findings with environmental monitoring initiatives and develop harmonized protocols to enable consistent comparisons across regions and production systems.

### 3.3. Heavy Metals

Heavy metals are among the most significant contaminants found in honey because they are persistent, mobile in the environment, non-biodegradable, and capable of bioaccumulating in biological systems. Unlike many organic pollutants, heavy metals do not break down chemically and can remain in environmental matrices for long periods, entering ecological and food chains through various contamination pathways. Since honeybees forage widely and constantly interact with soil, air, water, nectar, pollen, and plant surfaces, honey is increasingly seen as an effective bioindicator of environmental contamination and ecosystem health [21,35]. Exposure occurs when contaminants from atmospheric emissions or polluted soils are transferred into floral resources and then incorporated into hive products during foraging [11,22,23]. Consequently, honey bees and hive products are often used to monitor contamination patterns of pesticides, trace metals, polycyclic aromatic hydrocarbons, and dioxin-related compounds, especially in regions affected by human activities [35].

The presence of heavy metals in honey is primarily associated with anthropogenic activities, although natural geochemical factors can also contribute to regional differences in elemental levels. Major sources of contamination include industrial emissions, fossil fuel burning, vehicle traffic, mining, metallurgical processes, agricultural fertilizers, pesticides, wastewater discharges, and atmospheric deposits [4,20,35]. These pollutants can settle on plants or accumulate in soil, potentially contaminating nectar, pollen, and water that bees collect. This facilitates the transfer of toxic elements into hive products and ultimately into honey [11,22,23].

Several metallic elements have been frequently identified in honey, including lead (Pb), cadmium (Cd), chromium (Cr), nickel (Ni), copper (Cu), zinc (Zn), manganese (Mn), aluminum (Al), arsenic (As), and mercury (Hg). Their levels vary greatly depending on environmental factors, industrialization, land use, and pollution sources [35,36]. Table 1 summarizes key studies examining heavy metal contamination in honey from various regions.

As shown in Table 2, substantial geographical variability exists in the occurrence of heavy metals in honey. Lead (Pb) and cadmium (Cd) were the most frequently detected toxic elements, especially in samples from industrialized and urban areas. Higher levels typically came from industrial emissions, traffic, mining, and atmospheric inputs. Conversely, essential trace elements such as zinc, copper, manganese, and iron occurred at varying levels, influenced by floral source and environmental factors [36,37,38,39,40].

Additional evidence supports the role of honey as a sensitive biomonitoring matrix for detecting environmental metal contamination. Studies have shown that honeybees are unable to detect or avoid environmentally relevant concentrations of toxic metals, including arsenic, lead, and zinc, during foraging. As a result, contaminated food sources are readily consumed, facilitating the transfer of these metals into hive products. These findings further emphasize the value of honey as a bioindicator of environmental metal exposure [41].

From a toxicological standpoint, heavy metals are especially concerning because prolonged exposure can cause cumulative harmful effects in humans. Long-term consumption has been linked to oxidative stress, DNA damage, endocrine disruption, neurotoxicity, kidney problems, heart issues, immune system weakening, metabolic irregularities, and cancer risks [4,38,39]. Specifically, lead exposure is associated with neurological harm and developmental issues, cadmium with kidney damage and bone problems, mercury with neurotoxicity, and arsenic with cancer and heart-related illnesses. Due to these dangers, it is crucial to continuously monitor heavy metals in honey through analytical testing to ensure food safety, support environmental monitoring, and protect consumers [12,37,39].

### 3.4. Emerging Contaminants

Beyond traditional pollutants such as pesticides, veterinary antibiotics, and heavy metals, emerging contaminants in honey are drawing increased focus. Honeybees foraging across extensive areas constantly come into contact with vegetation, soil, water, and atmospheric particles, allowing environmental pollutants to enter hive products. Consequently, honey and other bee-derived substances are increasingly used as tools for biomonitoring environmental conditions and human-caused pollution [42,43]. Contaminants acquired during foraging can build up in honeybees and be transported back to the hive, where residues may be detected in honey, wax, pollen, and other bee products, demonstrating honey’s value as an environmental indicator.

Microplastics are a major focus among emerging pollutants because of their durability, widespread environmental presence, and possible risks to ecosystems and human health. These tiny plastic particles, less than 5 mm in size, come from the breakdown of larger plastics or are produced directly by industrial processes. They are found in the environment due to factors such as packaging degradation, synthetic fibers, industrial emissions, atmospheric transport, wastewater, and fragmented debris, enabling their dispersal across terrestrial, aquatic, and atmospheric environments [44,45,46]. Evidence increasingly shows that microplastics may contaminate food sources through atmospheric deposition, environmental transport, and processing, packaging, and storage.

Recent studies have detected microplastics in honey samples from multiple geographic regions, indicating that contamination is widespread rather than confined to isolated areas. Microplastic particles have been found in honey produced by both industrial and artisanal methods, with contamination levels shaped by local environmental factors, production techniques, and packaging materials. Commonly identified polymers include polyethylene (PE), polyethylene terephthalate (PET), polypropylene (PP), polyamide (PA), and ethylene-vinyl acetate (EVA), indicating contamination pathways from both environmental sources and technological processes. These results support the idea that contamination can arise not only from bee foraging and airborne particles settling on flowers but also during extraction, handling, processing, and packaging.

Current evidence suggests that honey is unlikely to be a major source of microplastics in individual diets. However, the repeated detection of microplastics raises concerns about long-term human exposure through food. Both experimental and observational studies indicate that microplastics may cause oxidative stress, inflammation, immune imbalances, endocrine disruptions, and cellular toxicity. They can also carry toxic substances and environmental pollutants [44,46]. Moreover, growing evidence indicates that exposure to these contaminants can affect honeybee health, including their physiology, behavior, immunity, and colony stability. This underscores the interconnected issues of environmental pollution, pollinator health, and food safety [43,46]. Overall, these findings highlight the importance of continuously monitoring emerging contaminants in honey and reinforce honey’s role as a bioindicator reflecting environmental pollution patterns [42,43,44,45,46,47].

From a food safety perspective, microplastic contamination is an emerging concern because, unlike traditional chemical residues, no standardized methods currently exist for extracting, identifying, quantifying, or assessing the risk of microplastics in honey. Their presence can stem from various sources of contamination, including atmospheric deposition, environmental exposure during foraging, beekeeping practices, processing, and packaging. Although analytical techniques such as μ-FTIR and Raman spectroscopy have enhanced our capacity to identify polymer particles, comparability of results across studies remains hindered by disparities in sample preparation, particle-size thresholds, contamination control procedures, and reporting standards. Furthermore, the health implications of consuming microplastics through honey remain incompletely understood. Future research endeavors should aim to establish harmonized analytical protocols, implement rigorous contamination controls, conduct detailed investigations of smaller particles, including nanoplastics, and assess their potential to carry other chemical contaminants. These existing gaps impede accurate exposure assessment and the formulation of evidence-based safety thresholds for honey [47,48,49].

The increasing presence of emerging contaminants highlights the need for broader monitoring efforts, enhanced analytical methods, and thorough risk assessments. As environmental pollution intensifies, the implementation of sensitive analytical techniques and multi-residue approaches will be essential for understanding contaminant behavior, assessing food safety risks, and protecting both environmental and human health.

### 3.5. Factors Influencing Contamination Levels

The presence and levels of contaminants in honey are shaped by a complex interplay of environmental, agricultural, geographical, and beekeeping factors. Since honeybees forage over large areas and constantly interact with various environmental compartments, the contamination found in honey mirrors both local pollution sources and wider ecological and human pressures [4,10,20].

Geographical location is a key factor influencing variability in contamination levels. Honey from industrialized or urbanized areas typically has higher pollutant concentrations than honey from rural, forested, or protected zones [4,10]. Sources such as industrial emissions, mining, traffic, waste disposal, and intensive agriculture significantly contribute to environmental pollution, affecting the chemical makeup of nectar, pollen, and water sources used by bees [20,48,49]. Numerous studies reveal distinct spatial patterns of contamination, particularly for heavy metals such as lead and cadmium, supporting honey’s role as an environmental biomonitoring tool [37,49].

Seasonal variation influences the presence and spread of contaminants. Agricultural practices, pesticide spraying schedules, weather patterns, and the availability of floral resources change throughout the year, impacting both when and how strongly contaminants are encountered [25,50]. For instance, pesticide residues are more commonly found during peak spraying periods, and variations in flowering plants can affect how environmental pollutants are transferred into hive products [16]. Additionally, weather factors such as rainfall, temperature, humidity, and wind can affect pollutant deposition, environmental stability, and movement [16].

Agricultural intensity and land-use practices significantly affect honey contamination. The methods, timing, and frequency of applying pesticides, fertilizers, irrigation, and crop management influence how pollutants enter ecosystems and ultimately reach hive products [24]. Highly intensive farming systems often have higher pesticide residues and decreased floral diversity, which can increase honeybees’ exposure to pollutants [16,24]. Additionally, environmental contamination from agrochemicals can cause accumulation of heavy metals and persistent organic compounds in nearby ecosystems.

Beekeeping management practices significantly influence variability in contamination levels. Factors such as the use of veterinary drugs, antibiotics, acaricides, hive treatments, feeding supplements, storage conditions, and hive hygiene can affect the presence of residues in honey [3,31]. Incorrect application of veterinary products, inadequate withdrawal periods, or poor post-harvest handling may raise contamination risks and compromise product quality. Moreover, variations in national regulations and apicultural practices may lead to regional differences in contaminant levels [3].

Also, the botanical and floral sources of honey further influence variability in contamination. Different plant species have unique abilities to absorb and accumulate contaminants from soils, atmospheric depositions, and water sources. As a result, honey’s composition and contaminant levels can vary with floral origin, local plant diversity, and environmental exposure conditions [9,24].

Ultimately, the rising presence of emerging contaminants such as microplastics, pharmaceutical residues, and long-lasting industrial pollutants complicates contamination profiles and food safety evaluations [6,42,43,44]. These pollutants highlight the ever-changing landscape of environmental pollution and emphasize the importance of ongoing monitoring strategies that account for multiple contamination pathways and evolving environmental threats.

Overall, contaminant levels in honey are influenced by a complex interplay of factors, including environmental quality, agricultural practices, apiculture methods, climate variability, and geographic features. Recognizing these factors is crucial for understanding contamination patterns, enhancing monitoring efforts, complying with regulations, and protecting both environmental and human health. The various contamination routes outlined above show that honey can contain a broad spectrum of chemical contaminants stemming from agriculture, beekeeping, environmental pollution, and emerging human-made sources. Table 2 provides a clear summary of the main types of contaminants, their sources, potential health impacts, and common analytical methods.

**Table 2:** Major contaminants detected in honey, their sources, health implications, and analytical determination methods.

Contaminant Class	Representative Compounds	Main Sources	Potential Health Effects	Main Analytical Methods	References
Pesticides	Neonicotinoids, organophosphates, carbamates, pyrethroids, organochlorines	Agricultural spraying, contaminated nectar, pollen and water sources	Neurotoxicity, endocrine disruption, oxidative stress, reproductive disorders, carcinogenicity	LC-MS/MS, GC-MS/MS	[24,25,26,27,28]
Veterinary antibiotics	Tetracyclines, sulfonamides, macrolides, fluoroquinolones, aminoglycosides, amphenicols	Apicultural treatments, contaminated hive matrices, improper withdrawal periods	Antimicrobial resistance, allergic reactions, public health concerns	LC-MS/MS, UHPLC-MS/MS	[29,30,31,32,33,34]
Acaricides	Amitraz, coumaphos, fluvalinate, flumethrin	*Varroa destructor* control treatments	Residue accumulation in hive products, chronic exposure risks, contamination of honey and wax	LC-MS/MS, GC-MS	[24,34]
Heavy metals and metalloids	Pb, Cd, As, Hg, Cr, Ni, Al	Industrial emissions, mining activities, road traffic, fertilizers, atmospheric deposition	Neurotoxicity, nephrotoxicity, carcinogenicity, cardiovascular and metabolic disorders	ICP-MS, ICP-OES, AAS	[35,36,37,38,39,40,41]
Polycyclic aromatic hydrocarbons (PAHs)	Benzo(a)pyrene, chrysene, benzo(a)anthracene, benzo(b)fluoranthene	Industrial emissions, fossil fuel combustion, urban pollution, atmospheric deposition	Genotoxicity, mutagenicity, carcinogenicity	HPLC-FLD, GC-MS/MS	[38,43]
Persistent organic pollutants (POPs)	PCBs, PBDEs	Industrial pollution and long-range atmospheric transport	Bioaccumulation, endocrine disruption, chronic toxicity	GC-MS/MS	[42]
Pyrrolizidine alkaloids	Echimidine, lycopsamine, retrorsine, senecionine	Nectar and pollen from toxic plant species	Hepatotoxicity, genotoxicity, carcinogenicity	LC-MS/MS	[50]
Microplastics	PE, PET, PP, PA, EVA	Atmospheric deposition, environmental contamination, processing and packaging materials	Oxidative stress, inflammatory responses, immune disruption, cellular toxicity	μ-FTIR, Raman spectroscopy, Py-GC-MS	[44,45,46,47]
Mycotoxins	Aflatoxins (AFB1, AFB2), ochratoxin A, patulin, zearalenone, sterigmatocystin	Fungal contamination of pollen, nectar and environmental matrices	Hepatotoxicity, nephrotoxicity, carcinogenicity	UHPLC-ESI-MS/MS, LC-MS/MS	[19]

## 4. Analytical Techniques for Contaminant Detection in Honey

Detecting and measuring contaminants in honey is challenging due to its complex composition and the low concentrations of these substances. The high sugar content and diverse organic and inorganic compounds can interfere with measurement methods, making it difficult to accurately identify contaminants [1,12,48]. Recently, significant progress has been made in developing selective analytical techniques capable of detecting a wide range of contaminants, including pesticide residues, veterinary drugs, heavy metals, mycotoxins, and environmental pollutants. These improvements are primarily driven by the growing need for food safety monitoring and stricter regulatory standards established by national and international authorities [6,16]. Modern analytical workflows typically combine effective sample preparation with advanced instrumental techniques.

Chromatography, frequently combined with sensitive detectors, remains essential for contaminant analysis, while spectrometry offers valuable supplementary data for identifying and quantifying particular compounds. Continuous improvements in analytical instruments have increased detection sensitivity, enabling the identification of contaminants at trace and ultra-trace levels [48,51].

**Figure 10:**
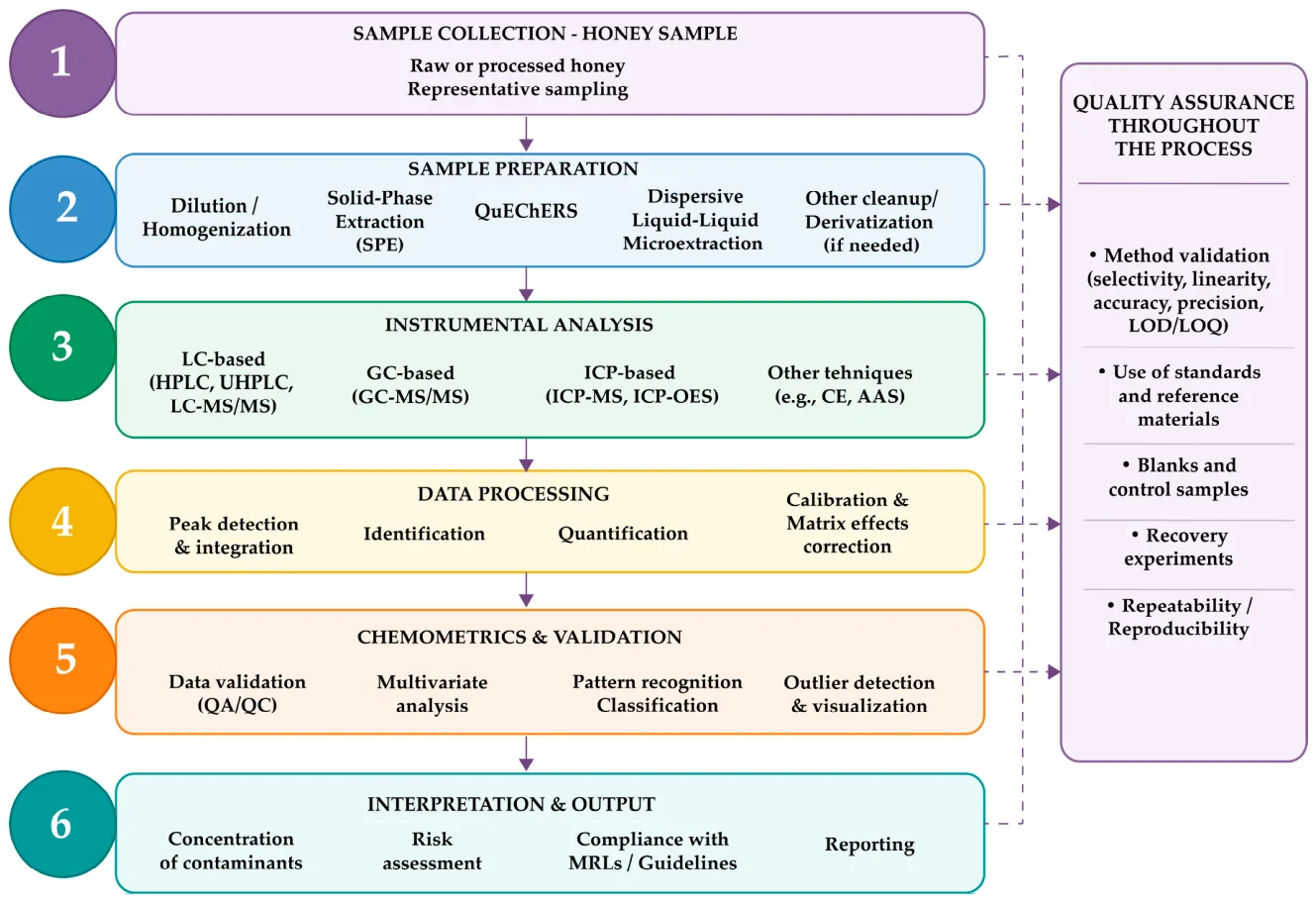
General analytical workflow for the determination of contaminants in honey.

Analytical laboratories face the challenge of detecting multiple contaminants within a single sample. To address this, significant efforts have been devoted to developing multi-residue methods capable of identifying compounds with diverse physicochemical properties. Such techniques improve analytical efficiency and reduce operational costs [16,48]. An important trend in honey analysis is the implementation of standardized validation procedures, which improve result reliability and comparability of results. Key parameters such as selectivity, recovery, accuracy, precision, detection and quantification limits are now fundamental elements of modern analytical methods. Using validated techniques helps generate datasets that support regulatory decisions and risk assessments [6,12]. The general workflow commonly applied for contaminant analysis in honey is summarized in Figure 10.

### 4.1. Chromatographic Techniques

Chromatographic techniques remain among the most used methods for honey analysis owing to their high selectivity, sensitivity, and ability to separate complex mixtures. The elevated sugar content and the presence of minor constituents—such as phenolic compounds, volatile substances, organic acids, pesticides, antibiotics, and other contaminants—render honey a challenging matrix that necessitates effective separation.

In honey research, chromatographic techniques such as high-performance liquid chromatography (HPLC), ultra-high-performance liquid chromatography (UHPLC), gas chromatography (GC), and chromatography coupled with mass spectrometry (LC-MS/MS and GC-MS) are used. These methods facilitate the identification and quantification of compounds at both high and low concentrations with high precision and reproducibility.

In addition to contaminant analysis, chromatographic fingerprinting combined with chemometric techniques has become an effective approach for honey classification and authenticity assessment. This methodology enables the discrimination of unifloral honeys based on HPLC and PTR-MS chemical profiles [52]. Rather than relying on a limited number of marker compounds, chromatographic fingerprinting captures the overall chemical composition of honey, generating characteristic profiles that can be analyzed using multivariate statistical methods such as principal component analysis (PCA), cluster analysis, and discriminant analysis. These approaches enable reliable classification of honeys by botanical origin and support the detection of mislabeling, adulteration, and fraudulent practices. Moreover, chromatographic fingerprints provide detailed information on compositional differences associated with floral source, geographical origin, environmental conditions, and processing methods, making them a valuable tool for honey quality control [52,53].

Mass spectrometry combined with chromatographic methods is essential for the analysis of certain types of honey, such as manna honey. It facilitates the identification of sugars, amino acids, proteins, phenolic compounds, volatile fractions, contaminants, and toxins. When coupled with high-resolution mass spectrometry, these techniques significantly enhance analytical capabilities, enabling detailed structural elucidation of compounds in complex honey samples. They have proven indispensable for identifying chemical markers associated with botanical and geographical origins and for elucidating differences in the compositions of nectar and manna honey. Characterization of minor constituents, including oligosaccharides, certain amino acids and phenolic derivatives, provides valuable information for authenticity verification and quality assessment. In addition, mass spectrometry platforms enable simultaneous detection of contaminants and natural bioactive compounds, providing a comprehensive assessment of honey’s nutritional quality [53].

Liquid chromatography is particularly valuable for the analysis of phenolic acids and flavonoids in honey due to their low concentrations and high structural diversity, which require sophisticated extraction, separation, and detection methods [54]. Phenolic compounds are key bioactive constituents of honey, mainly responsible for its antioxidant, anti-inflammatory, antimicrobial, and health-promoting properties. Contemporary liquid chromatography techniques, especially when combined with diode-array detection or mass spectrometry, enable precise identification. Recent innovations in sample preparation, solid-phase extraction, and multidimensional chromatography have further improved the efficiency of phenolic profiling. As a result, liquid chromatography has established itself as an essential tool for investigating the relationships among phenolic content, botanical origin, antioxidant activity, and the authenticity of honey samples [54].

#### 4.1.1. Liquid Chromatography–Tandem Mass Spectrometry (LC-MS/MS)

Liquid chromatography with tandem mass spectrometry (LC-MS/MS) is regarded as one of the most effective analytical methods for detecting contaminants in honey. Its high sensitivity, selectivity, and ability to analyze multiple compounds simultaneously make it ideal for monitoring pesticide residues, veterinary drugs, mycotoxins, plant toxins, and emerging environmental contaminants at trace and ultra-trace concentrations. Consequently, LC-MS/MS is a standard technique used in food safety laboratories and regulatory monitoring efforts globally [13,37,38,39].

The advanced resolving power of contemporary LC-MS/MS systems enables precise quantification of analytes in the complex honey matrix, thereby reducing matrix-related interferences and enhancing analytical accuracy. This significance is accentuated by honey’s high sugar content and diverse natural compounds, which can complicate contaminant detection [13,37]. LC-MS methods are widely used to detect naturally occurring toxins, such as pyrrolizidine alkaloids derived from certain nectar-producing plants. The presence of these compounds in commercial honey underscores the need for ongoing monitoring using highly sensitive analytical techniques to ensure consumer safety [50].

Recent methodological advances have expanded the use of LC–MS/MS for the determination of emerging contaminants, including per- and polyfluoroalkyl substances (PFAS). A validated UHPLC–MS/MS method has been successfully developed for quantifying PFAS in honey, delivering excellent analytical performance, very low detection limits in the ng/mL range, and high analytical accuracy. These findings further demonstrate the suitability of LC–MS/MS for monitoring persistent environmental pollutants that may contaminate honey through polluted ecosystems and agricultural activities [55].

In addition to chemical pollutants, LC-MS/MS is used to investigate emerging forms of contamination associated with environmental pollution. Recent studies have identified microplastic contamination in honey and have demonstrated increased interest in understanding the transfer of anthropogenic pollutants through food chains. These findings emphasize the necessity of highly sensitive analytical techniques capable of detecting emerging contaminants at trace levels [56]. Advanced UHPLC-MS/MS methodologies have also been developed to simultaneously determine nicotine and multiple mycotoxins in honey. These multi-residue methods offer high sensitivity, reliable quantification, and reduced analysis time, making them particularly attractive for routine quality-control applications and regulatory monitoring programs [19].

Beyond contaminant detection, liquid chromatographic techniques are increasingly employed to verify food authenticity and uncover food fraud. Non-targeted HPLC-UV fingerprinting combined with chemometric analysis has demonstrated excellent effectiveness in identifying honey adulteration with sugar syrups. Classification accuracy rates are reported to be nearly 100%, and quantitative models can accurately assess the level of adulteration in commercial samples. These findings highlight the value of chromatographic fingerprinting as a complementary quality-control method [57].

While gas chromatography remains the preferred technique for many volatile pesticide residues, liquid chromatography combined with mass spectrometry is becoming increasingly important for detecting polar and thermally unstable pesticides that are difficult for GC methods to identify. Consequently, the development of multi-residue workflows has significantly enhanced the capacity to monitor contaminants in honey and various bee products. Moreover, liquid chromatography is valuable for characterizing bioactive compounds in honey. LC-DAD-ESI/MS methods have successfully determined flavonoid profiles and phenolic compounds, supporting compositional analysis, botanical classification, and authenticity checks. When combined with multivariate statistical analysis, these techniques provide valuable insights into honey’s source and quality characteristics [58,59].

Overall, LC-MS/MS and other liquid chromatographic methods are essential for honey analysis today. Their ability to simultaneously detect contaminants, authenticity indicators, and natural bioactive compounds greatly improves food safety evaluations and quality control.

#### 4.1.2. Gas Chromatography–Mass Spectrometry (GC-MS)

While liquid chromatography-tandem mass spectrometry (LC-MS/MS) is highly effective for analyzing polar and thermally sensitive compounds, gas chromatography–mass spectrometry (GC-MS) continues to be the preferred method for detecting volatile and semi-volatile contaminants. Due to its superior separation capabilities and high sensitivity, GC-MS is extensively employed in the detection of pesticide residues, environmental pollutants, and volatile markers pertinent to honey quality and authenticity [58,60].

Pesticide residues are among the most significant contaminants examined by gas chromatography, due to their extensive agricultural use and potential transfer to bee products. Multi-residue GC-MS methods offer high accuracy, with recoveries from 76% to 95%, and detection limits below 0.01 mg/kg. These features enable reliable detection of multiple pesticide classes in a single analysis, aiding routine food safety monitoring [58]. The use of GC-MS has considerably enhanced our understanding of variations in pesticide contamination among honey samples from different regions. Research from diverse production zones demonstrates notable differences in residue concentrations, underscoring the influence of local agricultural practices, pest control strategies, and environmental factors. These findings further substantiate honey’s utility as a biomarker for assessing regional environmental pollution and agricultural impact [26].

Special attention has been given to persistent organochlorine pesticides due to their environmental persistence, propensity for bioaccumulation, and potential health hazards. Advanced GC-MS techniques offer a sensitive and dependable method for detecting these pesticide residues in honey, providing essential data on compounds that may persist in ecosystems long after their usage has been restricted or prohibited. The detection of these residues in honey underscores the enduring impact of environmental contamination and highlights the necessity for ongoing monitoring [59,60].

In addition to GC-MS, other gas chromatographic techniques have been used to examine persistent environmental pollutants. For example, gas chromatography paired with electron capture detection (GC-ECD) has proven effective in identifying organochlorine pesticides, revealing contamination patterns that may pose risks to environmental and human health [60].

The integration of HS-SPME-GC-MS with multivariate statistical techniques and artificial neural networks has yielded highly promising results. Research indicates that volatile compound fingerprints can reliably identify honey adulterated with high-fructose corn syrup, even at low levels. These methodologies provide a swift and reliable alternative to traditional authenticity testing and offer valuable insights into the composition and quality of honey [61]. Therefore, GC-MS remains an essential analytical technique in honey research. Its ability to detect pesticide residues, persistent environmental pollutants, and volatile authenticity markers renders it a versatile method that complements liquid chromatography and is integral to comprehensive honey quality evaluation.

#### 4.1.3. Multi-Residue and Advanced Chromatographic Approaches

The increasing diversity of contaminants in honey has driven the development of multi-residue analytical methods that detect multiple compounds with varying physical and chemical properties in a single analysis. Since pesticide residues, veterinary drugs, mycotoxins, environmental pollutants, and naturally occurring toxins can all be present at trace levels, current methods focus on achieving broad detection capabilities while ensuring high sensitivity, accuracy, and efficiency.

Achieving these objectives requires not only advanced chromatographic instrumentation but also effective sample preparation. The extraction step plays a decisive role in improving analyte recovery, minimizing matrix effects, and ensuring reliable quantification, especially when analyzing complex matrices like honey [32,33]. Among recent methods, single-drop microextraction combined with high-performance liquid chromatography has demonstrated effective detection of pesticide residues such as fipronil, while significantly reducing solvent use in line with green analytical chemistry principles [32].

The importance of sample preparation is further demonstrated by comparative studies evaluating QuEChERS, liquid–liquid extraction, and other miniaturized extraction techniques. These studies reveal that the choice of extraction method significantly impacts recovery rates, matrix interference, and overall analytical results, underscoring the importance of tailoring sample preparation to the specific analytes and honey matrix properties [32].

Once efficient extraction is complete, advanced chromatographic systems paired with tandem mass spectrometry enable simultaneous detection of multiple contaminants in a single analysis. These integrated methods greatly enhance laboratory efficiency while achieving low detection limits and good recoveries for pesticides, veterinary drugs, mycotoxins, and other environmental pollutants [55,58].

The same analytical platforms have been extended beyond contaminant detection. Non-targeted chromatographic fingerprinting, paired with chemometric analysis, provides specific chemical profiles that aid in honey authentication, adulteration detection, and botanical origin classification [57,59]. The use of multivariate statistical tools enhances data interpretation and classification precision. Recent applications involving elemental profiling show that these analytical methods have a wider scope, including honey characterization and environmental monitoring [59,62].

These developments collectively demonstrate how chromatographic methods are continually evolving into comprehensive analytical platforms capable of contaminant detection, authenticity verification, and quality assessment within a single integrated workflow.

### 4.2. Spectrometric Techniques

Spectrometric techniques are essential in honey analysis, particularly for identifying elemental composition, trace metals, and inorganic contaminants. In contrast to chromatographic methods, which primarily target organic compounds, spectrometric approaches provide direct insights into the mineral content of honey and potential toxic elements. Consequently, these methods are widely employed in food safety testing, environmental monitoring, authenticity verification, and quality assurance.

The elemental composition of honey is influenced by factors such as botanical origin, soil properties, climate, and human activities. Consequently, elemental profiling is a significant method for assessing environmental contamination and validating geographical origin and authenticity. When combined with chemometric analysis, spectrometric data facilitate the identification of specific elemental patterns and improve sample classification [63,64,65,66].

Among the available techniques, inductively coupled plasma–mass spectrometry (ICP-MS) and atomic absorption spectroscopy (AAS) are the most commonly employed methods for elemental analysis in honey. Although they differ in sensitivity and analytical capabilities, both techniques provide essential data for monitoring contaminants, conducting environmental assessments, and ensuring quality control [67,68,69].

#### 4.2.1. Inductively Coupled Plasma–Mass Spectrometry (ICP-MS)

Inductively coupled plasma–mass spectrometry (ICP-MS) is regarded as a key method for elemental analysis in honey due to its high sensitivity, low detection limits, and ability to analyze multiple elements at once. These features make it ideal for detecting trace metals and inorganic contaminants, aiding food safety assessments and authenticity testing of honey and other bee products [40,63]. Besides contaminant detection, ICP-MS provides detailed elemental fingerprints that reveal information about honey’s composition and origin. Levels of elements such as Pb, Cd, As, Ni, Cu, Zn, Mn, and Sr are strongly influenced by soil properties, botanical factors, geographic location, and human activities. As a result, elemental profiling is a valuable tool for assessing environmental exposure and verifying geographical and botanical origins [63,70].

The analytical potential of ICP-MS is further enhanced when combined with chemometric techniques like principal component analysis (PCA), cluster analysis, and linear discriminant analysis (LDA). These approaches help interpret complex datasets, enhance sample classification, and identify specific elemental markers linked to various production regions, resulting in highly reliable models for honey authentication and traceability [63].

ICP-MS also plays an important role in environmental pollution studies. Large-scale investigations have revealed significant spatial variations in toxic elements, particularly Pb, Cd, and As, linking contamination patterns to industrial activities, traffic emissions, agricultural practices, and other human activities. These results reinforce the idea that honey can serve as a bioindicator, providing insights into environmental health and contaminant spread in ecosystems [12].

Combining ICP-MS with chemometric analysis has considerably enhanced the value of elemental profiling in honey research. This integrated approach not only enables the determination of elemental composition but also supports authenticity assessment, geographical origin discrimination, contaminant detection, and evaluation of environmental impacts, providing a comprehensive strategy for honey characterization [40,63].

#### 4.2.2. Atomic Absorption Spectroscopy (AAS)

Atomic absorption spectroscopy (AAS) remains a popular method for detecting heavy metals in honey, especially in routine labs and environmental monitoring. While it is less sensitive than ICP-MS, AAS offers dependable measurements, straightforward operation, and affordability, making it a practical choice for evaluating elemental contamination in honey [71].

AAS has been widely used to detect potentially toxic elements like Pb, Cd, Cr, Ni, Cu, and Zn in honey, which can originate from atmospheric deposition, industrial emissions, agricultural activities, contaminated soils, and other environmental sources. Therefore, measuring these metals helps assess both food safety and environmental quality in honey-producing regions [12,40].

Studies performed on honey collected from urban, industrial, and agricultural regions have revealed considerable variations in elemental concentrations, reflecting differences in local environmental conditions and pollution sources. These findings further support the use of honey as a valuable bioindicator capable of monitoring contaminant accumulation and evaluating the effects of human activities on ecosystems [12,40].

In addition to environmental monitoring, Atomic Absorption Spectroscopy (AAS) plays an important role in routine quality assurance by ensuring that toxic metal levels in food comply with regulatory standards. Ongoing monitoring of heavy metal levels aids in consumer risk assessment and identifies environmental factors that may compromise honey safety. Recent studies have validated AAS’s effectiveness for this purpose, demonstrating its reliability in measuring trace elements in honey sourced from contaminated regions [71].

Although ICP-MS offers higher sensitivity and a wider range of elements, AAS is still a dependable and straightforward technique. Its reliability and lower operational costs make it a preferred choice for food safety testing, environmental monitoring, and regular honey quality checks [71].

### 4.3. Sample Preparation Techniques

Sample preparation is vital in honey analysis due to the honey matrix. The high sugar levels, along with proteins, organic acids, pigments, and other natural compounds, may interfere with analytical measurements and affect contaminant detection. As a result, effective extraction and cleanup procedures are essential to minimize matrix effects and achieve precise analytical results [48]. Selecting a sample preparation method depends on the target analytes and the analytical technique used. Organic contaminants, such as pesticides, antibiotics, and mycotoxins, need selective extraction procedures that enhance analyte recovery and minimize co-extracted matrix components. Conversely, elemental analysis typically requires complete sample digestion prior to instrumental measurement, especially when utilizing ICP-MS for multi-element analysis [40,63].

Recent developments have shifted sample preparation toward greener, faster, and more efficient analytical methods. Deep eutectic solvent (DES) systems have gained popularity as sustainable alternatives to traditional organic solvents, enabling effective extraction of flavonoids and other bioactive compounds while lowering solvent use and environmental impact [64]. At the same time, modern methodologies such as QuEChERS, dispersive solid-phase extraction (d-SPE), solid-phase microextraction (SPME), dispersive liquid–liquid microextraction (DLLME), and sugaring-out liquid–liquid extraction have improved extraction efficiency, minimized matrix interferences, and enhanced compatibility with advanced chromatographic and mass spectrometric techniques [72].

The increasing need for greater selectivity has driven the creation of new sorbent materials. Nanoparticle-assisted extraction enhances analyte recovery and purification by boosting sorbent surface area and selectivity, especially benefiting protein fingerprinting and honey authenticity research [65]. As analytical methods advance, optimized sample preparation remains crucial for producing dependable data to identify contaminants, verify authenticity, and ensure quality in honey and other bee products.

#### 4.3.1. Quick, Easy, Cheap, Effective, Rugged, and Safe (QuEChERS)

Among the sample preparation techniques currently used in honey analysis, QuEChERS (Quick, Easy, Cheap, Effective, Rugged, and Safe) has become one of the most widely adopted approaches for extracting pesticide residues and other organic contaminants. Originally designed for pesticide detection in food matrices, this method combines solvent extraction, salting-out partitioning, and dispersive solid-phase cleanup to ensure efficient analyte recovery while reducing matrix interferences. These features make QuEChERS especially suitable for honey analysis, given its complex composition and high sugar content [32,48].

One of the main advantages of QuEChERS is its compatibility with multi-residue analysis. The method can be adapted for the simultaneous extraction of compounds with different physicochemical properties and is therefore routinely combined with LC-MS/MS and GC-MS to determine pesticides, veterinary drugs, mycotoxins, and other contaminants [32].

Multiple studies have shown that QuEChERS-based protocols achieve high analyte recoveries, good reproducibility, and lower matrix effects, making them suitable for routine monitoring and regulatory purposes. Recently, QuEChERS has been effectively combined with advanced LC-MS/MS techniques for the simultaneous detection of mycotoxins and other contaminants, offering high sensitivity, reliable quantification, and enhanced laboratory efficiency [19].

The combination of simplicity, flexibility, and analytical performance has established QuEChERS as one of the most important sample preparation techniques for modern honey contaminant analysis [32,48].

#### 4.3.2. Solid Phase Extraction (SPE)

Although QuEChERS has become a popular method for multi-residue analysis, other sample preparation techniques remain important depending on the analytical goals and target compounds. For example, solid-phase extraction (SPE) is commonly used for sample cleanup, analyte preconcentration, and matrix simplification before instrumental analysis. SPE is especially useful for detecting trace contaminants in complex samples like honey. It relies on selectively retaining analytes on a solid sorbent and then eluting them in a controlled manner, thereby reducing matrix interferences, increasing analyte recovery, and boosting detection sensitivity. These features make SPE highly compatible with chromatographic techniques such as LC-MS/MS and GC-MS [48].

SPE can be used to detect a variety of contaminants, including pesticides, veterinary drugs, antibiotics, and other organic pollutants. Its ability to enhance method reproducibility and reduce detection limits has made SPE a key sample-preparation method for food safety checks and regulatory compliance [48]. Recent innovations in sorbent materials, especially nanostructured adsorbents and functionalized extraction phases, have further improved the selectivity and effectiveness of SPE methods. These developments enable the collection of high-quality analytical data to identify contaminants, verify authenticity, and assess honey quality [65].

#### 4.3.3. Liquid–Liquid Extraction (LLE)

Before the widespread adoption of techniques such as QuEChERS and SPE, liquid–liquid extraction (LLE) was one of the most commonly used approaches for isolating contaminants from food matrices. Despite the emergence of more efficient and environmentally friendly alternatives, LLE continues to be employed in certain analytical applications, particularly for method development, comparative studies, and validation purposes [49].

The principle of LLE is based on the partitioning of analytes between two immiscible liquid phases, typically an aqueous phase and an organic solvent. Although relatively simple, the technique often requires larger solvent volumes, multiple extraction steps, and longer processing times compared with modern extraction methodologies. These limitations have contributed to its gradual replacement by techniques offering higher efficiency and lower environmental impact [49].

Nevertheless, LLE remains an important reference method in analytical chemistry and is frequently used as a benchmark for evaluating the performance of newer extraction procedures. Comparative studies have demonstrated that while modern approaches generally provide superior recoveries, reduced matrix effects, and improved operational efficiency, LLE can still yield satisfactory analytical results when properly optimized [32,49].

Taken together, QuEChERS, SPE, and LLE illustrate the evolution of sample preparation strategies in honey analysis. The transition from conventional solvent-based extraction methods toward more selective, efficient, and sustainable approaches reflects the broader trend in chemistry toward improved analytical performance and reduced solvent consumption.

### 4.4. Rapid Screening and Spectroscopic Techniques

Rapid screening methods are increasingly recognized as useful tools for honey analysis because they offer quick, affordable, and non-destructive options compared to traditional lab techniques. Their ability to deliver rapid initial results with minimal sample preparation makes them ideal for routine quality checks, authenticity verification, and large-scale monitoring. Confirmatory testing can then be carried out using chromatographic or spectrometric techniques.

The growing concern about emerging contaminants underscores the need for rapid analytical methods to detect pollutants that might previously have been missed. Microplastics, in particular, have garnered significant attention due to their widespread presence in the environment and their potential to move through food chains. Their detection in honey from various regions underscores the need for more effective analytical techniques and standardized monitoring protocols to accurately assess their presence and potential food safety risks [56].

Spectroscopic methods have become the most widely investigated rapid screening tools for honey authentication because they generate characteristic chemical fingerprints with minimal sample preparation. When combined with chemometric analysis and machine learning algorithms, these techniques provide highly accurate classification models while considerably reducing analysis time. Among them, Fourier transform infrared (FTIR) spectroscopy has received particular attention due to its ability to rapidly characterize the chemical composition of honey. Recent studies integrating FTIR spectra with machine learning algorithms have achieved classification accuracies approaching 100% in detecting honey adulteration, highlighting the potential of artificial intelligence-assisted spectroscopy for rapid quality assessment [66]. The application of deep learning, particularly convolutional neural network (CNN)-based models, has further improved analytical performance by enabling the simultaneous detection of multiple adulterants while maintaining high sensitivity and specificity [67].

Other vibrational spectroscopic methods have demonstrated similar potential. Raman spectroscopy provides detailed molecular fingerprints that can distinguish honey by botanical origin, harvest time, and composition. When paired with chemometric analysis, prediction accuracies over 97% have been achieved, highlighting its effectiveness for authentication research [57,69].

Simple optical techniques have shown promising analytical capabilities. UV–Vis spectroscopy, when combined with multivariate statistical analysis, reliably distinguishes between genuine and adulterated honey, providing a quick and cost-effective option for initial quality checks [67]. Likewise, visible–near-infrared spectroscopy (Vis-NIRS), paired with partial least squares (PLS) regression and linear discriminant analysis (LDA), has achieved high classification accuracy, making it suitable for routine authenticity and quality control [59,68].

The integration of spectroscopic techniques with chemometric methods has greatly improved the efficiency and reliability of rapid honey analysis. These techniques offer dependable, non-destructive, and economical options that supplement traditional analytical techniques for detecting contaminants, verifying authenticity, and assessing quality.

## 5. Chemometric and Data Analysis Approaches

As analytical techniques such as chromatography, spectrometry, and spectroscopy yield increasingly complex data, chemometric analysis has become vital to contemporary honey research. These techniques generate extensive datasets that traditional statistical methods struggle to interpret effectively. Chemometric tools facilitate the extraction of meaningful insights from such complex data, aiding in honey authentication, geographical origin tracing, quality evaluation, and contaminant detection [1,24,62].

Chemometric methods are especially useful because honey composition depends on many factors, including botanical origin, location, climate, environmental contamination, and processing methods. As a result, multivariate statistical approaches are often used to detect patterns, reduce dimensionality, and improve sample classification.

Among unsupervised techniques, principal component analysis (PCA) remains the most popular method for exploring data. PCA lowers data dimensionality while maintaining key sources of variation, enabling visualization of similarities and differences among honey samples. It has been successfully applied to elemental, chromatographic, and spectroscopic datasets to distinguish honey by geographical origin, botanical source, and chemical composition. Additionally, PCA identifies the variables that most significantly contribute to sample differentiation [62,63].

Prediction methods such as partial least squares discriminant analysis (PLS-DA) and linear discriminant analysis (LDA) are now commonly used in authenticity studies. These techniques excel at distinguishing genuine honey from adulterated samples and classifying them by botanical and geographical origin [63]. Incorporating chromatographic fingerprinting with chemometric analysis has further enhanced detection accuracy, enabling precise identification and quantification of sugar syrup adulteration [57,59,73].

The rapid development of spectroscopic techniques has further expanded the role of chemometrics. FTIR, NIR, Vis-NIRS, Raman, and UV–Vis produce unique spectral signatures that, when analyzed using multivariate statistical methods, enable accurate classification of honey and help verify its authenticity without extensive sample preparation [71,72,73].

Recently, machine learning algorithms such as artificial neural networks (ANN), support vector machines (SVM), random forest models, and convolutional neural networks (CNNs) have greatly enhanced the predictive accuracy of chemometric models.

Beyond traditional chemometric methods, recent advances in artificial intelligence are expanding the analytical tools for assessing honey quality and safety. Machine-learning and deep-learning models can combine complex chromatographic, spectroscopic, and elemental data, uncovering nonlinear relationships often missed by standard multivariate techniques. Specifically, approaches like artificial neural networks, random forests, support vector machines, and deep-learning architectures show growing promise in identifying contamination patterns, detecting adulteration, classifying geographical and botanical origins, and interpreting high-dimensional analytical fingerprints. These methods could also enable the concurrent assessment of multiple safety-related factors and support rapid screening processes. Nevertheless, practical use is still limited by the need for large, representative datasets, variability in analytical platforms and preprocessing, lack of extensive external validation, and challenges in transferring models across laboratories. Therefore, future efforts should aim to improve predictive accuracy while developing robust, interpretable, and externally validated AI models that are suitable for routine honey quality and safety checks. These methods effectively handle complex non-linear data, automatically select important features, and improve classification performance, especially in research on honey adulteration and authenticity verification [66,67].

Another important development is the use of data fusion strategies, which integrate information from various analytical platforms into a single dataset. Merging chromatographic, spectroscopic, and elemental data enhances model robustness, boosts classification accuracy, and offers a more complete profile of honey’s composition and quality [62].

The continuous integration of chemometric methods with advanced analytical techniques and artificial intelligence has substantially expanded the possibilities for honey analysis, offering reliable tools for verifying authenticity, geographically tracing origins, monitoring contaminants, and assessing quality.

Although their analytical principles and target compounds differ, the techniques reviewed here encounter similar challenges in honey analysis. Honey’s complex, sugar-rich composition can impede measurements, making proper sample preparation, extraction, cleanup, and matrix effect management crucial [1,12,48]. Improvements in chromatographic and spectrometric tools have significantly enhanced sensitivity and selectivity, allowing detection of contaminants at trace and ultra-trace levels [48,51]. The growing need to identify multiple contaminants with diverse properties has driven the development of multi-residue methods, boosting efficiency and cutting costs [16,48]. Nonetheless, issues such as method validation, inter-method comparability, matrix effects, and protocol harmonization remain vital for dependable honey analysis [6,12]. Table 3 offers a comparative summary of the main analytical techniques, highlighting their key applications, shared challenges in honey analysis, addressed problems, and ongoing difficulties.

## 6. Food Safety and Regulatory Aspects

The presence of contaminants in honey raises significant concerns regarding food safety and consumer protection. Although honey is generally regarded as a natural and beneficial food product, contamination by pesticides, veterinary drug residues, heavy metals, environmental pollutants, and other undesirable substances may compromise its safety and quality. Because honey is frequently consumed across all age groups, including vulnerable populations such as children and the elderly, even low contaminant concentrations may contribute to adverse health effects with long-term exposure [2,74].

Within the European Union, the production, commercialization, and quality control of honey are regulated through a comprehensive legislative framework designed to ensure consumer protection and fair trade practices. The legal definition, composition requirements, and labeling rules for honey are set out in the Honey Directive, recently updated by Directive (EU) 2024/1438 [73]. In addition, honey is subject to the general principles of European food law established by Regulation (EC) No 178/2002, which provides the legal basis for food safety risk assessment, traceability, and consumer protection throughout the food chain [75].

The control of contaminants in honey is further supported by specific European regulations concerning pesticide residues, official food controls, and monitoring programs. Regulation (EC) No 396/2005 establishes maximum residue levels (MRLs) for pesticides in food products, including honey, while Regulation (EU) 2017/625 provides the framework for official controls performed by competent authorities to verify compliance with food safety legislation [76,77]. Furthermore, technical guidance documents and monitoring programs developed at the European and national levels aid the implementation of risk-based surveillance strategies for honey and other bee products [78,79].

Regulatory authorities, including the European Food Safety Authority (EFSA), the Codex Alimentarius Commission, and national agencies, play a key role in evaluating contaminants, setting safety standards, and coordinating monitoring activities. These efforts support the ongoing assessment of potential risks associated with honey consumption and help maintain high food safety standards across international markets [16].

### 6.1. Maximum Residue Limits (MRLs)

Maximum residue limits (MRLs) are among the principal regulatory tools used to control contaminant levels in food products and protect consumers from excessive exposure to potentially hazardous substances. MRLs define the highest legally permitted concentration of a specific residue in food and are established based on toxicological evaluations, dietary exposure assessments, and good agricultural practices. Within the European Union, pesticide MRLs are regulated under Regulation (EC) No 396/2005 and are regularly reviewed to reflect new scientific evidence and changes in agricultural practices [76].

For honey, MRLs are especially important because bees may be exposed to pesticides through contaminated nectar, pollen, water, or environmental deposition. Numerous monitoring studies have shown that most pesticide residues detected in honey remain below established regulatory limits, indicating a generally low risk to consumers. Nevertheless, occasional exceedances have been reported, particularly in areas with intensive agricultural activity and extensive pesticide use, underscoring the need for continuous monitoring and regulatory oversight [21,51].

The establishment and evaluation of pesticide MRLs in honey present specific analytical and regulatory challenges due to the unique characteristics of the honey matrix and the foraging behavior of bees. Consequently, dedicated European guidance documents have been developed to support harmonized approaches for residue assessment and risk evaluation in honey and other apicultural products [78].

In contrast to pesticides, the regulation of veterinary drug residues in honey is generally more restrictive. Within the European Union, the use of antibiotics in beekeeping is largely prohibited, and no specific MRLs have been established for many substances. As a result, the detection of antibiotic residues may lead to product non-compliance and market restrictions. This regulatory approach reflects concerns regarding antimicrobial resistance, food safety, and consumer confidence in apicultural products [3,16].

The implementation of MRL legislation is supported through official monitoring programs conducted by competent authorities at both European and national levels. In Romania, these activities are coordinated by the National Sanitary Veterinary and Food Safety Authority (ANSVSA), which participates in surveillance and control programs designed to ensure compliance with food safety requirements and protect public health [79].

### 6.2. Heavy Metals and Safety Thresholds

Heavy metals, alongside pesticide residues and veterinary drugs, are key contaminants monitored in honey because of their environmental persistence, tendency to bioaccumulate, and possible human toxicity. Reviews on honey safety highlight that contaminants such as heavy metals, pesticides, antibiotics, microorganisms, and other pollutants can compromise honey quality and pose health risks if proper monitoring is not in place [80]. Food safety monitoring systems further emphasize the importance of contaminant surveillance. RASFF notifications reveal that contamination is a recurring problem in the international honey trade, underscoring the necessity for ongoing control measures and regulatory oversight across the entire production chain [81].

Among inorganic contaminants, particular attention is given to lead (Pb), cadmium (Cd), arsenic (As), mercury (Hg), chromium (Cr), and nickel (Ni). These elements are of concern because they are not biodegradable and may accumulate in environmental compartments and food chains. Recent studies have demonstrated that environmental pollution significantly influences heavy metal concentrations in honey, with contamination levels often reflecting local industrial, agricultural, and urban activities [82]. The presence of heavy metals should be viewed within the larger framework of environmental pollution. Studies across various areas have simultaneously found trace metals and pesticide residues in honey, highlighting the complexity of contamination pathways and the importance of comprehensive food safety evaluations [83].

Heavy metals can contaminate honey via polluted soils, atmospheric fallout, industrial emissions, mining, traffic, and farming [12]. Evidence from Romania supports this link. Research in regions affected by mining and human activities has shown elevated levels of toxic elements in environmental samples and honey, confirming that local pollution sources affect honey quality and safety [84,85].

Regulatory contaminant monitoring extends beyond environmental pollutants to include residues of veterinary medicinal products, particularly antibiotics. Their presence can lead to non-compliance with European legislation and pose additional food safety risks. Multiple studies analyzing honey’s mineral and metal content have shown that it can serve as a useful indicator of environmental quality. The levels of both essential and toxic elements in honey often mirror local ecological conditions, endorsing its use in environmental monitoring [12,40,60,86]. Honey bees forage over extensive areas and regularly interact with soil, water, nectar, pollen, and airborne particles. As a result, honey is increasingly viewed as a bioindicator of environmental contamination. Research on heavy metal profiles shows that elemental composition can reveal pollution sources and help verify the authenticity and origin of honey [87].

While most studies indicate that heavy metal levels stay within international safety standards, long-term exposure remains a concern because these toxins can accumulate in the human body over time. Persistent dietary intake has been associated with adverse health effects such as neurotoxicity, kidney damage, developmental problems, endocrine disruption, and an increased cancer risk [12]. Therefore, risk evaluations highlight the importance of continuous monitoring, particularly in areas with high levels of environmental pollution, to help minimize health risks associated with honey consumption [88].

### 6.3. Risk Assessment and Consumer Exposure

Detecting contaminants in honey does not necessarily indicate a direct health threat. Therefore, food safety assessments now often use risk-based methods that combine contaminant levels with consumption habits, toxicological reference points, and exposure scenarios tailored to specific populations. These approaches help generate more accurate estimates of health risks from honey intake and assist in making informed regulatory choices [21,89]. Dietary exposure is typically estimated by comparing contaminant intake to toxicological reference values, including the acceptable daily intake (ADI), tolerable daily intake (TDI), hazard quotient (HQ), and hazard index (HI). These indicators help determine whether contaminant concentrations detected in honey are likely to pose health concerns under typical consumption conditions [21,82,89].

Most studies suggest that contaminant levels in honey are unlikely to pose a significant health risk to the general population when consumed in normal amounts. Nonetheless, exposure can vary greatly depending on factors such as location, environment, sources of contamination, and individual eating habits [21,90]. Although the overall risk remains low, specific groups such as children, pregnant women, the elderly, and high honey consumers may face higher relative exposure due to differences in body weight, metabolism, and diet, even if contaminant levels stay within regulatory limits [21,91].

Risk assessment now covers a wider spectrum of environmental pollutants beyond traditional contaminants. Polycyclic aromatic hydrocarbons (PAHs), for instance, have gained attention due to their persistence, carcinogenic properties, and widespread presence in the environment. Finding PAHs in honey, especially from urban and industrial regions, emphasizes honey’s effectiveness as a matrix for environmental biomonitoring [89].

At the same time, evaluating combined exposure to multiple contaminants remains a major challenge. Honey can simultaneously contain trace levels of heavy metals, pesticide residues, veterinary drugs, PAHs, and other pollutants. Although the concentrations of individual contaminants usually meet regulatory standards, the long-term cumulative or synergistic effects are poorly understood and often inadequately addressed in current risk assessments [6,21,90].

Advances in analytical techniques have enhanced exposure assessment by enabling detection of contaminants at even lower concentrations and facilitating the identification of emerging pollutants. As new toxicological data become available, regulatory agencies are compelled to regularly revise risk assessment frameworks and monitoring methodologies to adapt to evolving exposure scenarios [34,89,91]. Nevertheless, existing evidence indicates that honey remains a safe food for the general population when produced under appropriate environmental and apicultural conditions. Continuous monitoring and updated risk assessment methods are vital for protecting consumer health and ensuring adherence to evolving food safety standards [21,89,90,91].

### 6.4. Regulatory Challenges and Monitoring Systems

The globalization of honey markets, increasing complexity of supply chains, and growing consumer demand for premium and monofloral products have intensified the need for robust monitoring systems capable of detecting both contamination and fraud. Recent studies evaluating honey quality in Eastern Romania have highlighted the importance of continuous monitoring of physicochemical parameters, including moisture content, acidity, electrical conductivity, and mineral composition, as essential indicators of compliance with national and European quality standards. These investigations have also demonstrated that geographical origin, climatic conditions, harvesting year, and handling practices may significantly influence honey quality and therefore require continuous regulatory surveillance [92].

Recent evaluations of honey from Iași County reaffirm the critical role of systematic quality control programs. Comparative testing of raw and commercial honey samples revealed notable differences in physicochemical properties, mineral content, acidity, hydroxymethylfurfural levels, and other quality parameters associated with processing and storage. These results underscore the need for standardized analytical methods and robust monitoring systems to ensure product authenticity, safeguard consumers, and meet regulatory standards across the food supply chain [93].

Beyond routine quality checks, regulatory authorities face growing challenges in ensuring product standardization and compliance with legal quality standards. Recent studies on Romanian honey highlight the importance of monitoring physicochemical parameters, mineral content, and contaminants to comply with European legislation and strengthen quality assurance efforts. Additionally, correlations among mineral composition, physicochemical traits, and environmental influences underline the value of integrated analytical methods for evaluating quality, tracing origins, and verifying authenticity [94].

Another significant challenge involves economically motivated adulteration and honey fraud. Recent reviews indicate that advanced adulteration methods are becoming harder to identify with traditional analytical techniques alone. Global trade expansion, varying regulatory standards across countries, and the evolution of more sophisticated fraud tactics highlight the need for integrated, data-driven authentication systems. These systems should effectively combine spectroscopic, chromatographic, isotopic, and omics-based techniques within unified regulatory frameworks [95].

In addition to authenticity issues, monitoring programs should focus on environmental contamination and health risks. Research on mercury in honey shows that risk-based monitoring is crucial, especially in areas affected by industrial, agricultural, or urban pollution. While contaminant levels often stay below safety limits, ongoing surveillance is important since chronic exposure can lead to cumulative health impacts and long-term environmental effects [96]. Similar findings have been observed for other trace metals, including cadmium, chromium, copper, manganese, and zinc. Overall, health-risk assessments suggest that honey consumption poses low non-carcinogenic risks. Nonetheless, ongoing environmental pollution necessitates regular monitoring and periodic reassessment of consumer exposure, particularly in regions affected by human activities [97].

The concept of ecological safety has gained increasing attention in recent years. Modern monitoring now extends beyond single contaminants to include the combined effects of heavy metals, pesticides, veterinary drug residues, radionuclides, and other environmental stressors. Mathematical and multivariate models have been developed as effective tools for assessing the overall environmental safety of honey and pinpointing the contaminants that most significantly impact quality and pose health risks [98]. Likewise, research on health-risk assessment highlights the importance of combining physicochemical characterization, contaminant analysis, and toxicological indicators for thorough food safety evaluations. These methods provide a more accurate picture of consumer exposure and help inform decisions on food safety management and regulatory policies [99].

Emerging contaminants pose a new regulatory challenge. Recent reviews underscore the growing importance of tracking contaminant mixtures such as pesticides, persistent organic pollutants, pharmaceuticals, polycyclic aromatic hydrocarbons, and microplastics. These substances are not always fully regulated under current laws, leading to gaps that may necessitate updates in food safety policies, monitoring efforts, and analytical standards. Additionally, thorough evaluations of honey quality and related health risks highlight the need for ongoing regulatory adjustments to address newly identified contaminants and changing exposure conditions [100,101].

To address these issues, extensive monitoring systems have been implemented at both national and global levels. In the European Union, the Rapid Alert System for Food and Feed (RASFF) acts as a primary tool for swiftly sharing information about food safety problems and non-compliant products. This system enables authorities to detect emerging risks, coordinate responses, and stop unsafe products from reaching consumers. Analyses of RASFF alerts related to honey have repeatedly identified issues such as contamination, adulteration, mislabeling, and regulatory violations, underscoring the importance of ongoing monitoring and enforcement to sustain consumer trust and market transparency [16,81].

Future advances in honey safety management will increasingly depend on sophisticated analytical techniques, digital traceability systems, integrated databases, chemometric tools, and risk-based inspection strategies. When combined with harmonized legislation and strong international cooperation, these innovations will enhance regulatory authorities’ ability to detect contamination, fight food fraud, and protect consumers in a complex global market. Ensuring honey safety ultimately requires ongoing collaboration among researchers, laboratories, producers, regulatory agencies, and public health organizations to address both current and emerging food safety issues [73,75,76,77,78,79,95].

## 7. Challenges and Limitations

Although there have been significant improvements in analytical tools and contaminant detection, several challenges continue to limit the accurate assessment of honey’s safety, quality, and authenticity. These challenges arise from the complex nature of the honey matrix, the variety of contamination sources, differences in methods, and the growing sophistication of food fraud techniques.

One of the most significant analytical challenges is the complexity of honey’s chemical composition. Honey consists predominantly of sugars; it also contains proteins, enzymes, amino acids, organic acids, minerals, phenolic compounds, volatile substances, and various minor components. This complexity can cause matrix effects that hinder extraction efficiency, analyte recovery, signal strength, and precise quantification, especially when contaminants are present at trace or ultra-trace levels. Therefore, extensive sample preparation and highly sensitive analytical methods are often necessary for accurate results [1,30]. The variety of contamination sources further challenges contaminant assessment. The presence of pesticide residues, veterinary drug residues, heavy metals, and emerging contaminants depends on a range of environmental, agricultural, industrial, and beekeeping factors. Variations in geography, floral sources, climate, land-use practices, and local pollution can lead to significant differences among honey samples. This variability makes it difficult to identify consistent contamination patterns and hampers direct comparisons between studies from different regions [4,10].

Methodological inconsistencies are another key limitation. Variations in sampling strategies, extraction methods, instrumental techniques, quality assurance, and validation criteria can lead to significant differences in analytical results. Although standardized methods exist for many conventional contaminants, there are few harmonized approaches for many emerging contaminants and authenticity markers. This lack of standardization hampers comparability between laboratories and may impact the reliability of surveillance data and risk assessments [6,17].

The growing complexity of honey adulteration techniques presents additional challenges. Today’s food fraud strategies go beyond mere sugar additions, often employing advanced adulterants engineered to imitate the physicochemical, isotopic, and compositional traits of genuine honey. Consequently, traditional authenticity tests may fall short in their discriminatory power, necessitating the use of supplementary analytical methods, chemometric techniques, and multilayer authentication systems to identify increasingly sophisticated fraudulent schemes [102].

Environmental changes add another layer of uncertainty. Factors such as climate change, habitat degradation, biodiversity loss, and shifts in flowering patterns can markedly affect bee foraging, floral resource availability, and routes of exposure to environmental pollutants. These changes may alter honey’s composition and contamination profiles over time, posing challenges for long-term monitoring and necessitating adaptive surveillance strategies that accommodate evolving environmental conditions [103].

Economic and infrastructural limitations impact honey monitoring efforts globally. Techniques such as LC-MS/MS, GC-MS/MS, HRMS, ICP-MS, metabolomics, proteomics, and genomics offer high-performance analysis but often require costly equipment, specialized staff, and substantial operational expenses. As a result, access to these technologies varies significantly among countries and labs, leading to differences in monitoring capabilities and regulatory enforcement. Detecting and evaluating emerging contaminants is particularly challenging. Microplastics, pharmaceutical residues, persistent organic pollutants, and emerging environmental chemicals are receiving increasing scientific attention, but analytical methods for their detection are still developing. Additionally, many of these contaminants lack fully established regulatory guidelines and toxicological reference values, which restricts thorough risk assessment and informed regulatory decisions [34,71].

Another limitation relates to how cumulative exposure is evaluated. Current risk assessment methods typically examine individual contaminants, but honey often contains multiple classes of residues and environmental pollutants simultaneously. The interactions between these co-occurring contaminants—whether synergistic, additive, or antagonistic—are not well understood. This underscores the need for more comprehensive exposure models that can better represent actual consumption scenarios.

An important limitation of current honey safety assessments is that toxicological evidence mostly considers contaminants individually. However, consumers are often simultaneously exposed to mixtures of pesticide residues, potentially toxic elements, veterinary drugs, environmental pollutants, and emerging contaminants. Advances in analytical methods enable detection of such co-occurrence, but the toxicological significance of low-dose, chronic, and combined exposures remains underdeveloped. Therefore, special attention is needed for potential additive, synergistic, or antagonistic effects among different contaminant classes, especially for vulnerable populations and long-term dietary exposure [34]. Future research should combine realistic honey occurrence data with mixture-exposure assessments and mechanistic toxicology to determine whether low-concentration contaminant combinations can cause biological effects. This evidence will be crucial for shifting from mere contaminant detection to a more comprehensive, risk-based evaluation of honey safety [71,72,73,75,76,104].

Ultimately, honey quality assessment goes beyond just contamination. It also involves factors such as premature harvesting, insufficient maturation, poor storage conditions, processing methods, and packaging practices, all of which can significantly affect honey’s composition and quality indicators. Differentiating between natural compositional differences and contamination effects is complex and requires a multidisciplinary analytical approach that combines chemical, microbiological, physicochemical, and authenticity tests [104,105,106].

## 8. Future Perspectives

Future research on honey contamination, authenticity, and food safety will likely depend more on integrated analytical frameworks. These frameworks are essential to tackle the increasing complexity of environmental contamination and food fraud. As new contaminants appear and adulteration methods evolve alongside changing environmental conditions, analytical techniques must possess high sensitivity, selectivity, robustness, and sustainability.

One of the primary directions for future development is to expand multi-residue analytical methods to simultaneously detect multiple contaminant classes, including pesticides, veterinary drug residues, heavy metals, mycotoxins, pharmaceuticals, persistent organic pollutants, and microplastics. Combining advanced chromatography with high-resolution mass spectrometry is anticipated to significantly enhance contaminant detection, boosting both analytical effectiveness and laboratory throughput.

Future analytical developments should also strengthen the verification of honey authenticity. As adulteration methods become more sophisticated, there is a need for advanced tools that go beyond relying on a single marker. Combining metabolomics, proteomics, genomics, isotopic analysis, and chemometric modeling creates promising avenues for developing thorough authentication systems. These can detect subtle compositional changes and complex fraudulent activities. Implementing such approaches could greatly boost traceability, regulatory oversight, and consumer trust in global honey markets [107].

The principles of green analytical chemistry are anticipated to become more influential in future method development. Sustainable sample preparation methods, such as microextraction techniques, deep eutectic solvents, solvent-free extraction systems, and miniaturized platforms, can decrease solvent use, energy consumption, and waste generation without compromising analytical quality. These innovations support wider sustainability goals and environmental protection efforts [64].

Artificial intelligence (AI), machine learning (ML), and advanced data analytics are set to revolutionize honey research. Modern analytical tools produce large, complex datasets that often surpass the capabilities of traditional statistical methods. AI-driven algorithms can enhance pattern recognition, classification, contaminant prediction, authenticity checks, origin tracing, and fraud detection. Integrating omics technologies with machine learning models can significantly boost predictive accuracy and enable automated decision-making systems [66,67].

Another promising direction involves combining data from environmental, climatic, and biological monitoring efforts. Large datasets from surveillance programs, climate models, biodiversity surveys, and bee health tracking can offer important insights into contaminant behavior, exposure routes, and ecosystem impacts on honey quality. These integrated methods could enhance understanding of how environmental changes affect pollinator health and food safety [103].

Finally, future research should focus more on assessing cumulative exposure and mixture toxicology. As consumers are frequently exposed to multiple contaminants at once, understanding their potential synergistic effects and long-term health consequences is crucial for more accurate risk assessments. Creating integrated toxicological models that evaluate combined exposures could greatly enhance consumer safety and support evidence-based food safety policies [108,109].

In summary, future developments in honey safety will increasingly rely on the integration of artificial intelligence, emerging contaminant monitoring, and toxicological risk assessment. Artificial intelligence facilitates the analysis of complex data sets, while hazards such as micro- and nanoplastics require enhanced detection methods, standardized monitoring protocols, and a more profound understanding of their toxicological impacts. The amalgamation of these advancements with mixture-based and long-term exposure assessments can establish a more comprehensive framework for identifying novel risks and formulating evidence-based safety strategies for honey.

## 9. Conclusions

Honey serves as both an important food product and an environmental bioindicator, reflecting the quality of the ecosystems in which honeybees forage. Besides being a food product, honey serves as an important environmental bioindicator because its composition reveals the ecological conditions of honeybee foraging regions. The rise in intensive agriculture, pollution, industrial activities, and global trade has increased worries about honey’s quality, authenticity, and safety. Therefore, detecting and monitoring contaminants in honey has become crucial for modern food safety and environmental monitoring.

This review offers a thorough overview of current research on honey contamination, including sources, occurrence, analytical methods, chemometric techniques, and food safety considerations. It also features a bibliometric analysis to explore the development of scientific research, identify key thematic areas, and highlight emerging trends in honey contamination and authenticity. Additionally, the review critically examines recent progress in sample preparation, chromatographic, spectrometric, and rapid spectroscopic methods, spotlighting their importance in contaminant detection, authenticity verification, and quality evaluation. Integrating bibliometric mapping with a critical review of the literature provides a comprehensive view of current challenges, methodological innovations, and future directions in honey safety and environmental monitoring. Notable advancements have been achieved in analytical testing methods, with techniques such as LC-MS/MS, GC-MS, ICP-MS, and ICP-OES enhancing sensitivity, selectivity, and accuracy in contaminant detection. These techniques enable trace and ultra-trace analysis, multi-residue screening, and detailed chemical profiling. Furthermore, innovations in sample preparation—including QuEChERS-based protocols, microextraction, deep eutectic solvents, and nanomaterial-assisted extraction—have improved analytical performance while promoting more sustainable laboratory practices.

In parallel, the incorporation of chemometric methods, machine learning, artificial intelligence, and advanced statistical approaches has significantly expanded the analytical capabilities in contemporary honey research. These tools help interpret intricate datasets, refine contaminant classification and source tracing, and bolster authenticity verification, geographical origin tracking, and fraud detection. The combination of advanced instrumentation and data-driven analysis is expected to become increasingly vital in future food safety monitoring efforts.

Despite these advances, various challenges still exist. The intricate composition of honey, diverse sources of contamination, the absence of standardized testing methods for certain contaminant types, and limited access to advanced analytical tools all impede coordinated monitoring efforts. Moreover, toxicological assessments typically examine single contaminants, whereas real-world exposures involve complex chemical mixtures whose combined or synergistic effects are not yet fully understood.

Future research should prioritize developing comprehensive multi-residue analytical platforms capable of simultaneously detecting both conventional and emerging contaminants. Additionally, efforts should be made to harmonize analytical protocols, expand international monitoring networks, improve regulatory frameworks, and create globally comparable reference datasets. Special focus is needed on evaluating combined exposure risks, understanding long-term health effects, and studying how environmental changes affect contaminant levels in bee products.

Despite the substantial progress made in contaminant monitoring and analytical technologies, several challenges persist. These encompass the detection of emerging contaminants, the harmonization of analytical methodologies and monitoring programs, the evaluation of cumulative exposure to multiple contaminants, and the development of environmentally friendly, cost-effective, and high-throughput analytical strategies. Future research should further integrate advanced analytical techniques with chemometric tools and environmental monitoring to improve honey quality assessment, support regulatory decision-making, and promote consumer protection.

Ensuring honey safety requires an integrated approach that combines advanced analytical technologies, continuous environmental monitoring, effective regulatory oversight, sustainable agricultural practices, and responsible beekeeping management. Such coordinated efforts are crucial not only for protecting consumer health but also for preserving pollinator populations, supporting environmental sustainability, maintaining product authenticity, and strengthening confidence in honey throughout the global food supply chain.

## Figures and Tables

**Table 1:** Occurrence of heavy metals in honey from different geographical regions.

Study Area	Study Characteristics	Main Metals Detected	Key Findings	Reference
Romania	61 honey samples collected from eight Romanian geographical regions	K, Ca, Mg, Na, Mn, Zn, Cu, Cr, Pb, Cd, Li, Sr, Ni, Al	Significant regional variability in elemental composition was observed. Pb and Cd exceeded established safety limits in some samples, indicating potential anthropogenic contamination and environmental pollution.	[36]
Northern Italy (Lombardy)	57 honey samples collected between 2011 and 2022 from industrial and rural areas	Pb	Lead contamination was detected primarily in honey samples collected from industrial areas. Industrial combustion processes and road traffic were identified as probable sources of contamination.	[37]
Poland (urban apiaries)	35 honey samples collected from six apiaries located in urbanized areas	Zn, Cd, Pb	All samples contained detectable concentrations of Zn, Cd, and Pb. Lead exceeded permissible limits in some samples, whereas cadmium concentrations remained relatively low. Overall, contamination levels did not significantly compromise honey quality.	[38]
Calabria, Southern Italy	38 honey samples of different floral origins (wildflower, citrus, chestnut, honeydew, and erika) collected from beekeeping farms	Pb, Cd, As, Zn, Mn, Fe, Cu, Se, Al	All analyzed elements were detected. Pb concentrations exceeded the EU maximum residue limit (0.1 mg/kg) in most samples, although estimated dietary exposure remained low. The study confirmed the usefulness of honey as an environmental monitoring tool.	[39]
Northwest England	31 honey samples collected by citizen-scientist beekeepers across the Greater Manchester area	As, Cd, Cr, Cu, Pb, Zn, Ni	Honey reflected local contamination patterns associated with current and historical anthropogenic activities. Cd and Pb concentrations exceeded WHO/FAO guideline values in some areas, supporting the use of honey as a biomonitoring matrix.	[40]

**Table 3:** Comparative overview of analytical techniques applied to honey, their common analytical aspects, main achievements, and remaining challenges.

Analytical Technique	Main Applications/Target Analytes in Honey	Common Aspects When Applied to Honey	Main Achievements/Problems Addressed and Remaining Challenges
LC-MS/MS/UHPLC-MS/MS [16,32,33,48,55,58]	Pesticides, veterinary drugs, mycotoxins, and other organic contaminants	Efficient extraction and clean-up are required, while matrix effects associated with the complex honey matrix must be controlled. These techniques are particularly suitable for trace-level and multi-residue analysis.	Achievements: High sensitivity and selectivity and simultaneous determination of multiple contaminants with different physicochemical properties. Challenges: Matrix effects, optimization of extraction for diverse analytes, expansion of multi-residue coverage, and harmonization and validation of analytical protocols.
GC-MS/GC-MS/MS [48,51,55,58]	Pesticide residues, persistent environmental pollutants, and volatile or semi-volatile compounds	Appropriate sample preparation and chromatographic separation are essential to minimize interference from the honey matrix. Applicability is strongly dependent on analyte volatility and physicochemical properties.	Achievements: Sensitive detection and confirmation of pesticide residues and persistent pollutants and characterization of volatile compounds. Challenges: Sample preparation remains critical, applicability to non-volatile compounds is limited, and more efficient multi-contaminant workflows are required.
HPLC/UHPLC [32,52,53]	Pesticides and other organic compounds; chemical profiling and authenticity-related applications	Effective chromatographic separation is required because honey contains high sugar concentrations together with numerous minor organic constituents that may interfere with analysis.	Achievements: Reliable separation and quantification of target compounds and generation of chromatographic fingerprints useful for broader chemical characterization. Challenges: Matrix interference, analyte-dependent optimization, and the need for integration with advanced detection and data-processing approaches.
ICP-MS [12,40,63]	Trace metals and inorganic contaminants; elemental profiling	Careful sample preparation and contamination control are essential. Interpretation of elemental profiles must also account for geographical, botanical, and environmental variability.	Achievements: High sensitivity, low detection limits, and simultaneous multi-element determination; useful for contaminant monitoring and elemental fingerprinting. Challenges: High instrumental requirements, interpretation of geographical variability, and the need for comparable analytical and monitoring strategies.
AAS [12,40]	Heavy metals and potentially toxic elements, including Pb, Cd, Cr, Ni, Cu, and Zn	Appropriate sample preparation is required for reliable elemental determination, and results may reflect both direct food contamination and environmental exposure.	Achievements: Reliable and comparatively accessible determination of selected metals, particularly suitable for routine elemental monitoring. Challenges: Lower sensitivity and multi-element capability than ICP-MS and limited analytical throughput for broad elemental profiling.
FTIR/NIR/Vis-NIRS/Raman/UV–Vis spectroscopy [57,59,66,67,68,69]	Rapid compositional analysis, authenticity assessment, adulteration detection, and classification	Spectral fingerprints generally require chemometric or machine-learning processing to extract meaningful information and discriminate between samples.	Achievements: Rapid, non-destructive, and comparatively economical analysis with limited sample preparation and strong potential for classification. Challenges: Dependence on representative calibration datasets, model validation, reproducibility, and transferability between sample sets and instruments.
Chemometric and machine-learning approaches (PCA, LDA, PLS-DA, SVM, ANN, RF, CNN) [1,24,57,59,62,63,66,67]	Interpretation of chromatographic, elemental, and spectroscopic datasets; authentication, classification, geographical-origin assessment, and contaminant-related applications	Their performance depends on analytical data quality, appropriate preprocessing, representative datasets, and robust model validation.	Achievements: Improved interpretation of complex multidimensional datasets, pattern recognition, classification, and extraction of information not readily apparent from individual analytical variables. Challenges: Model robustness, external validation, transferability between laboratories and sample populations, and the availability of sufficiently large and standardized datasets.
Integrated multi-residue approaches [16,32,33,48,55,58]	Simultaneous determination of pesticides, veterinary drugs, mycotoxins, and environmental contaminants	These approaches require a balance between extraction efficiency, reduction in matrix effects, broad analyte coverage, sensitivity, and appropriate method validation.	Achievements: Simultaneous detection of multiple contaminant classes within a single analytical workflow, reducing analysis time and improving laboratory efficiency. Challenges: Simultaneous coverage of compounds with substantially different physicochemical properties and the harmonization and validation of broad-scope methods.

## Data Availability

No new data were created or analyzed in this study. Data sharing is not applicable to this article.

## Abbreviations

The following abbreviations are used in this manuscript:

AAS	Atomic Absorption Spectrometry
ADI	Acceptable Daily Intake
AI	Artificial Intelligence
ANN	Artificial Neural Network
ANSVSA	National Sanitary Veterinary and Food Safety Authority
CNN	Convolutional Neural Network
DES	Deep Eutectic Solvent
DLLME	Dispersive Liquid–Liquid Microextraction
DNA	Deoxyribonucleic Acid
EC	Electrical Conductivity
EFSA	European Food Safety Authority
EU	European Union
EVA	Ethylene-Vinyl Acetate
FTIR	Fourier Transform Infrared Spectroscopy
GC	Gas Chromatography
GC-ECD	Gas Chromatography–Electron Capture Detection
GC-MS	Gas Chromatography–Mass Spectrometry
HI	Hazard Index
HMF	Hydroxymethylfurfural
HPLC	High-Performance Liquid Chromatography
HPLC-UV	High-Performance Liquid Chromatography–Ultraviolet Detection
HQ	Hazard Quotient
HRMS	High-Resolution Mass Spectrometry
HS-SPME-GC-MS	Headspace Solid-Phase Microextraction–Gas Chromatography–Mass Spectrometry
ICP-MS	Inductively Coupled Plasma–Mass Spectrometry
ICP-OES	Inductively Coupled Plasma–Optical Emission Spectrometry
LC-DAD-ESI/MS	Liquid Chromatography–Diode Array Detection–Electrospray Ionization–Mass Spectrometry
LC-MS/MS	Liquid Chromatography–Tandem Mass Spectrometry
LDA	Linear Discriminant Analysis
LLE	Liquid–Liquid Extraction
ML	Machine Learning
MRL	Maximum Residue Limit
NADES	Natural Deep Eutectic Solvent
NIR	Near-Infrared Spectroscopy
NIRS	Near-Infrared Spectroscopy
PA	Polyamide
PAH	Polycyclic Aromatic Hydrocarbon
PCA	Principal Component Analysis
PBDEs	Polybrominated Diphenyl Ethers
PCBs	Polychlorinated Biphenyls
PE	Polyethylene
PET	Polyethylene Terephthalate
PFAS	Per- and Polyfluoroalkyl Substances
PLS	Partial Least Squares
PLS-DA	Partial Least Squares Discriminant Analysis
POPs	Persistent Organic Pollutants
PP	Polypropylene
PTR-MS	Proton Transfer Reaction–Mass Spectrometry
QuEChERS	Quick, Easy, Cheap, Effective, Rugged and Safe
RASFF	Rapid Alert System for Food and Feed
SPE	Solid-Phase Extraction
SVM	Support Vector Machine
TDI	Tolerable Daily Intake
UHPLC	Ultra-High-Performance Liquid Chromatography
UHPLC-MS/MS	Ultra-High-Performance Liquid Chromatography–Tandem Mass Spectrometry
UV	Ultraviolet
PRISMA	Preferred Reporting Items for Systematic Reviews and Meta-Analyses
GC-MS/MS	Gas Chromatography–Tandem Mass Spectrometry
UHPLC-ESI-MS/MS	Ultra-High-Performance Liquid Chromatography–Electrospray Ionization–Tandem Mass Spectrometry
PAHs	Polycyclic Aromatic Hydrocarbons
AFB1	Aflatoxin B1
AFB2	Aflatoxin B2
WHO	World Health Organization
FAO	Food and Agriculture Organization
